# Peptides Targeting GDNF Family Receptor Alpha 1 (GFRα1) Mimic Glial Cell Line-Derived Neurotrophic Factor (GDNF) Bioactivity

**DOI:** 10.1021/acs.jmedchem.5c03413

**Published:** 2026-05-08

**Authors:** Emily A. Atkinson, Tianyang Liu, Maria Fowler, Poppy O. Smith, Alethea B. Tabor, Christopher J. Morris, James B. Phillips, Rachael Dickman

**Affiliations:** 1 School of Pharmacy, 4919 UCL, 29-39 Brunswick Square, London WC1N 1AX, U.K.; 2 Centre for Nerve Engineering, 4919 UCL, 29-39 Brunswick Square, London WC1N 1AX, U.K.; 3 Department of Chemistry, 4919 UCL, 20 Gordon Street, London WC1H 0AJ, U.K.

## Abstract

Glial cell line-derived neurotrophic factor (GDNF) is a neuroprotective protein with widespread applications in regenerative medicine, though its clinical translation has been limited. Peptide mimetics have the potential to overcome the limitations associated with the translation of GDNF. Herein, peptides targeting the GDNF receptor GFRα1 were identified by screening a 12-mer linear peptide phage display library against GFRα1. Peptide hits were rationalized by computational alanine scanning mutagenesis studies to identify residues contributing to GDNF and GFRα1 binding. Four hit peptides were synthesized and tested for GFRα1 binding affinity. All peptides activated the downstream phosphatidylinositol 3 kinase signaling pathway in SH-SY5Y cells and increased cellular proliferation. The biological activity of two peptides matched that of recombinant GDNF, as measured by neurite outgrowth of primary dorsal root ganglion neurons. For the first time, we report monomeric GFRα1-targeting peptides that mimic the biological effects of GDNF, with potential applications in regenerative medicine.

## Graphical abstract

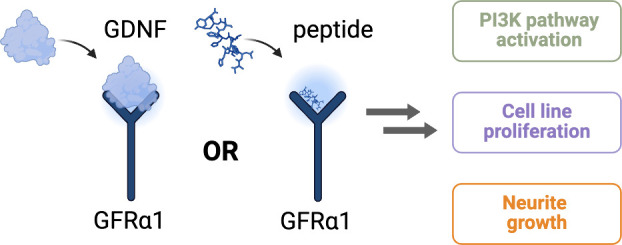

## Introduction

Growth factors are endogenous signaling proteins that bind to specific receptors on the cell surface, thereby activating intracellular signaling pathways that control biological processes. Various growth factors have the potential to be therapeutic candidates for regenerative medicine, with their ability to promote tissue repair and regeneration, promote neuron survival and axonal growth, as well as enhance angiogenesis.^1^

Glial cell line-derived neurotrophic factor (GDNF) is a 30.4 kDa naturally occurring neurotrophic factor. It is known for its ability to support dopaminergic neuron survival, and is vital for the development of the enteric nervous system and kidneys,^2,3^ as well as being crucial for spermatogenesis.^4^ GDNF has therefore been extensively explored for the treatment of Parkinson’s disease, showing potential in animal models and clinical trials.^5–9^ The therapeutic potential of GDNF extends beyond neurodegenerative disorders. It has been shown to promote axonal regeneration and neuroprotection following spinal cord and peripheral nerve injury, as well as facilitate wound healing and hair growth. The widespread applications of GDNF in regenerative medicine have been recently reviewed.^10,11^

GDNF is a member of the GDNF-family ligands (including neurturin, artemin and persephin). Each of these ligands exerts its biological effects by binding to the extracellular region of the rearranged during transfection (RET) tyrosine kinase receptor, in complex with its corresponding GDNF family receptor alpha (GFRα) coreceptor (GFRα1–4).^12–15^ The pro-region of GDNF is first proteolytically cleaved,^16^ then active, dimeric GDNF preferentially binds to two GDNF family receptor alpha 1 (GFRα1) receptors.^14,17^ The GDNF-GFRα1 complex then binds to two RET receptors, though GDNF itself does not directly bind to RET alone, promoting RET homodimerization and intracellular phosphorylation (Figure 1).^18^ This binding mechanism is understood to activate multiple intracellular signaling pathways, including the phosphatidylinositol 3 kinase (PI3K)-AKT pathway, the extracellular signal-regulated kinase-mitogen-activated protein kinase (ERK-MAPK) pathway, and Src kinase signaling, all of which contribute to neuroprotection.^19,20^ In cells that lack the RET receptor, such as Schwann cells, cortical neurons and hippocampal neurons, GDNF-GFRα1 can instead signal through the neuronal cell adhesion molecule (NCAM).^21^ Additionally, GDNF is also capable of signaling independently of RET and GFRα1 via the heparan sulfate chains on syndecan-3.^22^

**Figure 1:**
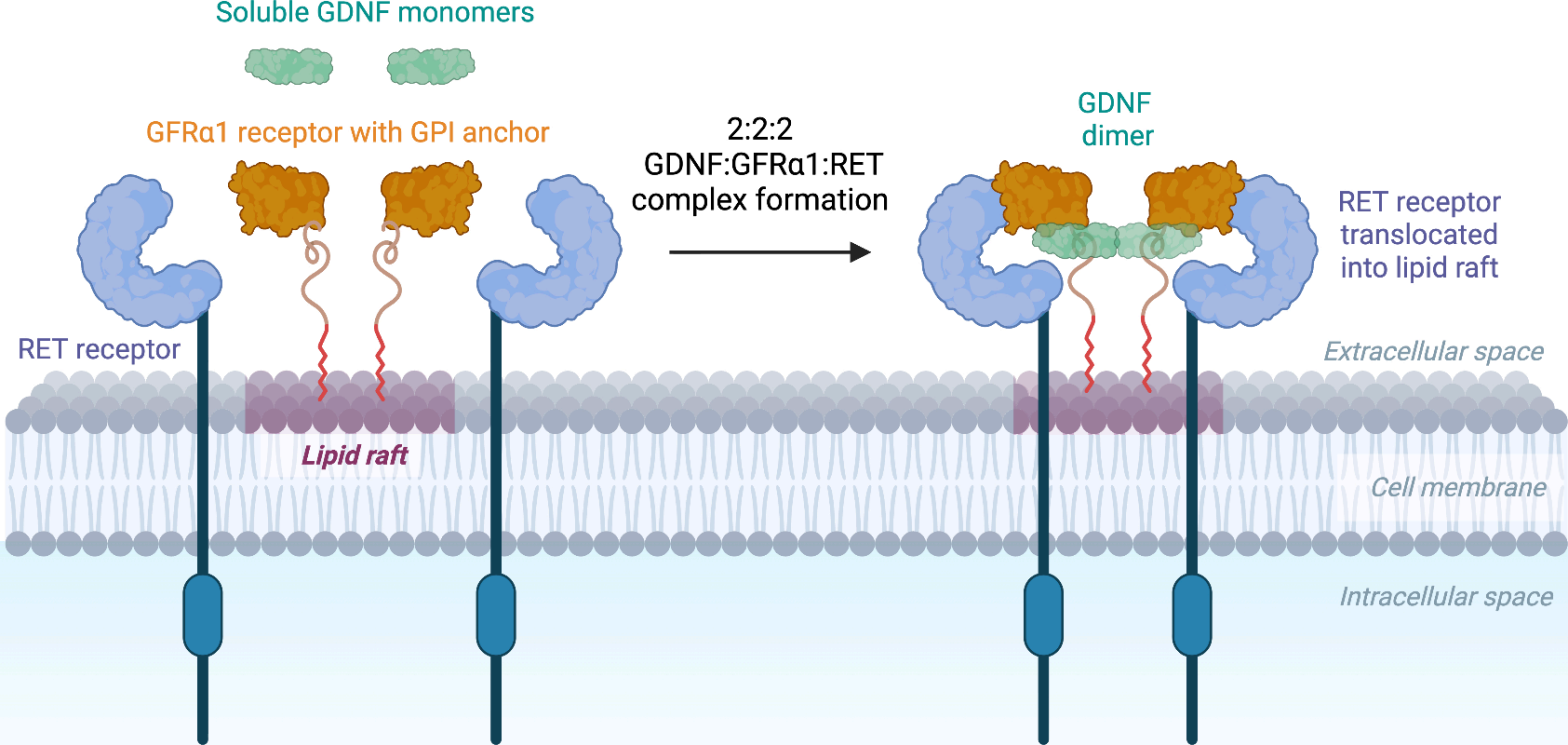
GDNF-GFRα1-RET binding mechanism. GDNF dimer forms a complex with two GFRα coreceptors that are glycosyl phosphatidylinositol (GPI)-anchored and are localized in lipid rafts on the plasma membrane of cells, including neurons.^23^ The complex binds to two transmembrane RET receptors, translocated into lipid rafts.

Due to its broad therapeutic potential, recombinant GDNF is a protein of interest for regenerative medicine. Despite this, the clinical translation of GDNF and other growth factors has been limited by diffusion from the target site leading to reduced efficacy and frequent dosing, barriers in delivery, immunogenicity, batch variability, and costly manufacture due to the expression of GDNF in mammalian cell cultures. Various modalities have been employed to overcome some of these issues, such as cell therapies, gene therapies, oligonucleotides, small molecule mimetics and peptide mimetics.^24–32^ For example, a series of small molecules which bind to GFRα1-RET have been developed which have demonstrated ability to protect dopaminergic neurons both *in vitro* and *in vivo*.^33,34^ Peptides have some key advantages over other mimetics, including low immunogenicity, improved pharmacokinetics, cheap and scalable production, and the potential for increased interaction with large receptor surfaces compared with small molecules.^35^

Peptide mimetics can mimic the biological activity of a growth factor, including binding to the same target receptor, or by activating the same downstream signaling cascades. Synthetic peptide mimetics of growth factors have potential to be used in place of recombinant growth factors, with a decrease in size and more favorable pharmacokinetic properties.^28,36,37^ Peptides have better tissue penetration, are easier to modify for improved stability, and have a more scalable and consistent method of synthesis, making peptide therapeutics more affordable and therefore, accessible. Few peptide mimetics of GDNF have been reported to date, with current peptide mimetics being derived from native growth factor sequences (DNSP-11, gliafin and artemin).^37–39^ Gliafin and artefin are peptide dendrimers and therefore lose some of the pharmacological advantages of a monomeric peptide mimetic. DNSP-11 is a peptide derived from the GDNF pro-domain which has shown therapeutic potential *in vitro* and *in vivo*.^40–42^ However, it is predicted to act by a different binding and signaling mechanism to GDNF, and does not activate RET.^38^

An alternative approach to identify peptide mimetics of GDNF is high throughput screening methods, for example utilizing mRNA and phage display libraries. Phage display can be used to identify potential peptide growth factor mimetics by screening genetically modified bacteriophages (phages) that express recombinant peptides on their surface coat proteins, against a target receptor.^43^ For example, phage display has previously been utilized successfully to identify brain-derived neurotrophic factor (BDNF) peptide mimetics that increased SH-SY5Y cell survival to the same extent as the recombinant growth factor.^44^ Commercial libraries of phage have been engineered to express up to 10^10^ unique peptide sequences for high-throughput screening. Following several rounds of biopanning, Illumina next-generation sequencing (NGS) can be used to identify the peptide sequences with affinity for the target receptor, which can then be synthesized and tested for target receptor binding and target activation.

Here, we report the first GFRα1-targeting monomeric peptides which mimic the biological action of GDNF. The peptides were discovered by screening a linear 12-mer phage display library against full-length human GFRα1. Four peptide hits were selected for further analysis based on the extent of library enrichment and sequence homology to GDNF. Synthetic peptides mimicked the pharmacological action of the GDNF protein by binding GFRα1, with nanomolar *K*_D_, and subsequently activated the downstream PI3K signaling pathway. Functional assays in cell cultures demonstrated that these peptides induced comparable SH-SY5Y cell proliferation and dorsal root ganglion (DRG) neurite outgrowth to that of GDNF.

## Results and Discussion

### Discovery of Peptides Binding to GFRα1 by Phage Display Screening the Ph.D-12 Library against His-Tagged GFRα1

The commercially available Ph.D-12 library contains approximately 1 × 10^9^ unique peptide sequences containing 12 randomized residues, expressed at the *N*-terminus of the minor coat protein (pIII) of M13 phage virions. This library was selected to identify peptide binders of GFRα1 with a 20-fold decrease in size compared to native dimeric GDNF, appropriate for a scalable synthesis. Three rounds of affinity-based biopanning were performed against the His-tagged target receptor (GFRα1), using magnetic capture (Figure 2A). Over three rounds of library screening, the output titer increased, consistent with a library enrichment of binding clones (Figure 2B). 2637 unique GFRα1-binding peptide sequences were identified in the unamplified round three screening output using Illumina NGS and downstream analysis using P3ANUT.^45^ Nonselective and parasitic sequences identified using Mimoscan were excluded.^46^ The top 20 remaining enriched sequences were ranked based on the number of counts (Table 1).

**Figure 2:**
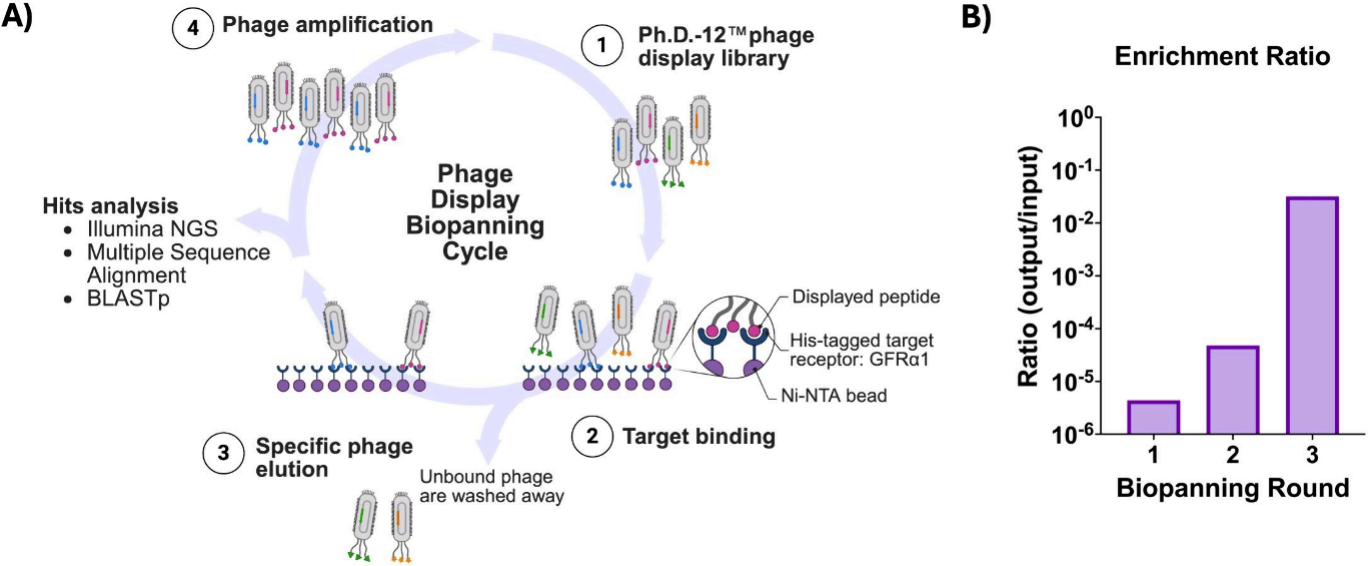
Phage display biopanning cycle with enrichment ratios. (A) Schematic of the phage display biopanning process, using affinity capture. (B) Enrichment ratio (unamplified phage output titer divided by input titer) for each round of biopanning.

**Table 1:** Top 20 Peptide Sequences Obtained from Phage Display^a^

rank	peptide sequence	name	peptide counts
1	TNSHHLHHSAQY	L12–1	76,133
2	RKQHAIPLIWPA	L12–2	70,221
3	SGPRHYHHLLEL		42,654
4	WWQKPHFHPLET		38,282
5	RTHSHHPMQYHL		28,998
6	GVGNHAHKLTWH		17,186
7	LPLHDHWHMRHA		16,097
8	GYQRHWTPVPHH		12,271
9	VVSPDMNLLLTN	L12–3	11,221
10	HLRHGQPSVFHA		9,338
11	TYTHHSSSQHYG		9,098
12	LPRHSTQPTHYH		8,269
13	QQRPYVQDLRLI	L12–4	7,930
14	HKVHRVNTWGGL		6,818
15	HLKIHHVRVDHM		5,640
16	KASGSPSGFWPS		5,542
17	VGFHHLTEHPHR		5,186
18	GPVRQHVPRHFH		4,432
19	SQQYALTNSTTN		4,149
20	HLRPVHHVASRH		4,101

a Peptides were ranked according to the peptide counts determined by Illumina NGS, after three rounds of biopanning. The four sequences named L12-1 to L12-4 were selected for synthesis and further analysis.

The top 20 ranked peptide sequences were compared with multiple sequence alignment. This showed low sequence similarity, with no conserved clusters of residues (Figure S2A). The frequency of His residues across these sequences was unexpectedly high, with most of the top 20 sequences containing His residues in a 1,3 or 1,6 pattern (55%, Figure S2B). This could indicate nonspecific binding of the peptides to the Ni-NTA beads used in the biopanning, although this was mitigated with negative selection.^47^ The two peptides with the highest number of sequencing counts were selected for synthesis (named L12–1 and L12–2, respectively), with only L12–1 encompassing a 1,3 His pattern.

The top 20 ranked enriched sequences were also compared to the native GDNF sequence using BLASTp. Peptides ranked at numbers 9 and 13 (Table 1), named L12–3 and L12–4 respectively, had high sequence homology to the two ‘finger regions’ of GDNF (approximately 42% and 33%, respectively) which are known to contribute to native GDNF-GFRα1 binding (Figure 3A,B).^48^ To determine if the key GDNF-GFRα1 binding interactions were likely to be maintained by L12–3 and L12–4, computational alanine scanning mutagenesis studies were conducted using BAlaS (Table S4).^49^ This analysis predicts the change in ΔΔG_binding_ when each amino acid is mutated to alanine, with residues being identified as hot spots when the ΔΔG_binding_ > 0.5 kJ/mol. Hot constellations were also identified using BAlaS which combines multiple alanine mutations to find which hot spots on GDNF cooperate in the binding pocket (Table S5). A hot constellation is identified when the ΔΔG_binding_ calculated when multiple residues are simultaneously mutated to alanine, is greater than the sum of the ΔΔG_binding_ individual values when mutated to alanine independently. Interestingly, some of the residues identified as binding hot spots in the BAlaS analysis were present in the same positions in L12–3 and L12–4 (PDB: 6Q2N,^48^ Figure 3C) and were also identified within most hot constellations. When considered together with experimental mutagenesis studies,^13^ this suggests that key interactions between GDNF-GFRα1 may indeed be mimicked by L12–3 and L12–4 and GFRα1 (Figure 3C,D). However, some residues which were predicted to be important for GDNF-GFRα1 binding, such as Tyr197 in finger 2 and Glu138 in finger 1 of GDNF, did not appear in the L12–3 and L12–4 sequences in the equivalent positions (boxed positions, Figure 3C). Despite the phage display library encompassing approximately 10^9^ peptide sequences, it is possible that these precise sequences did not appear in the library, or that the selected peptides form different interactions with the receptor than native GDNF. Given the homology of L12–3 and L12–4 to the two finger regions of GDNF, these two sequences were also selected for further analysis. To investigate whether the four selected peptides were likely to engage the GFRα1 receptor in a similar manner to GDNF, the interactions between the peptides and receptor were modeled using AlphaFold3.^50^ Though the interaction was not modeled with high confidence, all four peptides were predicted to bind to the same domain of GFRα1 as GDNF (Figure S3).

**Figure 3:**
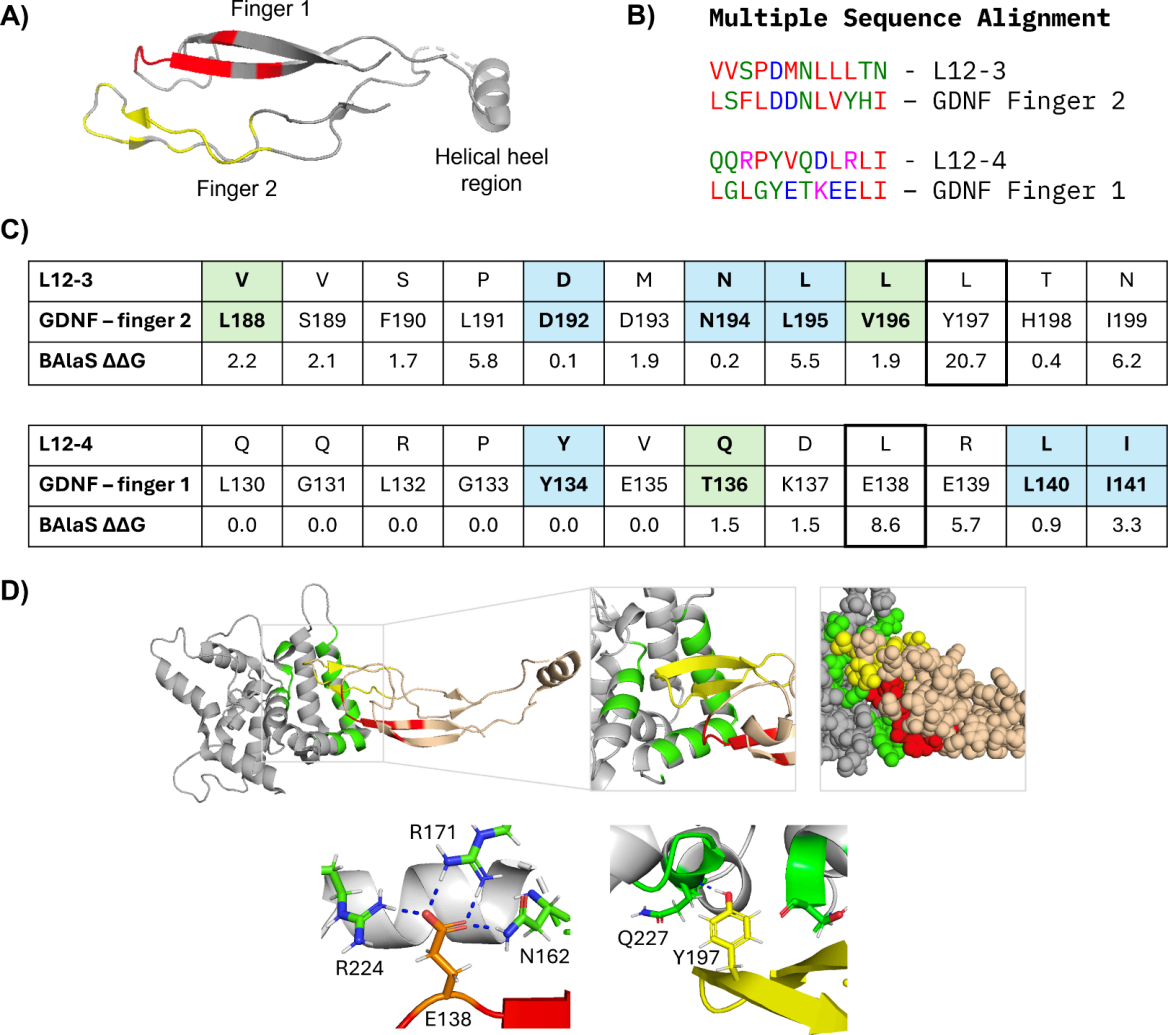
Comparing L12–3 and L12–4 peptide sequences to GDNF. (A) Crystal structure of GDNF (PDB: 6Q2N), highlighting the two finger regions of GDNF. Hot spots identified from computational alanine scanning mutagenesis experiments using BAlaS (hot spots are those with a ΔΔG_binding_ > 0.5 kJ/mol, when the stated amino acids were mutated to alanine)^49^ are highlighted on finger one (red) and finger two (yellow). (B) Multiple sequence alignment of L12–3 and L12–4 compared to the homologous regions of GDNF, highlighting common residues where red represents hydrophobic residues, blue represents acidic residues, magenta represents basic residues and green represents other polar residues. (C) Comparing peptide sequences L12–3 and L12–4 to the two finger regions of GDNF, including ΔΔG from computational alanine scanning experiments using BAlaS. Blue represents conservation of the amino acid between the native sequence and the phage display hit, and green represents amino acids with similar properties. (D) Crystal structure of GDNF (PDB: 6Q2N), highlighting the interactions between GFRα1 (gray) and GDNF (sand). Hot spots on GDNF which were identified using BAlaS are highlighted in red (finger 1) and yellow (finger 2). Residues in GFRα1 which are involved in interaction with GDNF are highlighted in green. The polar carboxyl group on the E138 side chain (GDNF, red) acts as a hydrogen bond acceptor, forming four interactions with N–H groups on the side chains of R224, R171 and N162 (GFRα1, green). The phenolic hydroxyl group on the side chain of Y197 (GDNF, yellow) points into the binding site on GFRα1, potentially acting as a hydrogen bond donor with the backbone carbonyl of Q227 (GFRα1, green).

### Peptides Selected by Phage Display Screening Imitate GFRα1 Binding and Biological Activity of GDNF

The four peptides selected (L12–1, L12–2, L12–3 and L12–4) were synthesized using solid-phase peptide synthesis (SPPS) for further biophysical and biological testing. All four peptides bound to the target receptor (GFRα1), confirmed with Surface Plasmon Resonance (SPR) kinetic affinity experiments. All peptides showed a concentration-dependent binding to His-tagged GFRα1, with nanomolar equilibrium dissociation constants (*K*_D_) calculated using steady-state affinity models (Figure 4, Figure S9, and Table S8). L12–1 bound to GFRα1 with a dissociation constant of approximately 55 nM. Peptide L12–2 had the highest affinity to the receptor, with a dissociation constant of ∼ 3 nM. Interestingly, this was not the top ranked peptide in the phage display experiments, or one of the peptides with homology to GDNF. BSA and L12–5, a peptide with lower expected affinity to GFRα1 identified from the phage display screening, were used as negative controls. No nonspecific interactions were observed between the immobilized peptides and BSA. A repeat binding experiment between L12–1 and Fc-tagged GFRα1 showed a *K*_D_ that was consistent with the His-tagged protein (Figure S10). All four peptides are potent and therefore potentially therapeutically useful. As expected, all peptides have a weaker affinity for GFRα1 than native GDNF (*K*_D_ 0.63 nM, determined with SPR).^51^ This is likely because the peptides are 20-fold smaller than dimeric GDNF, and therefore may be too small to cover the whole of the GFRα1 binding interface interacted with by the two finger regions of native GDNF.

**Figure 4:**
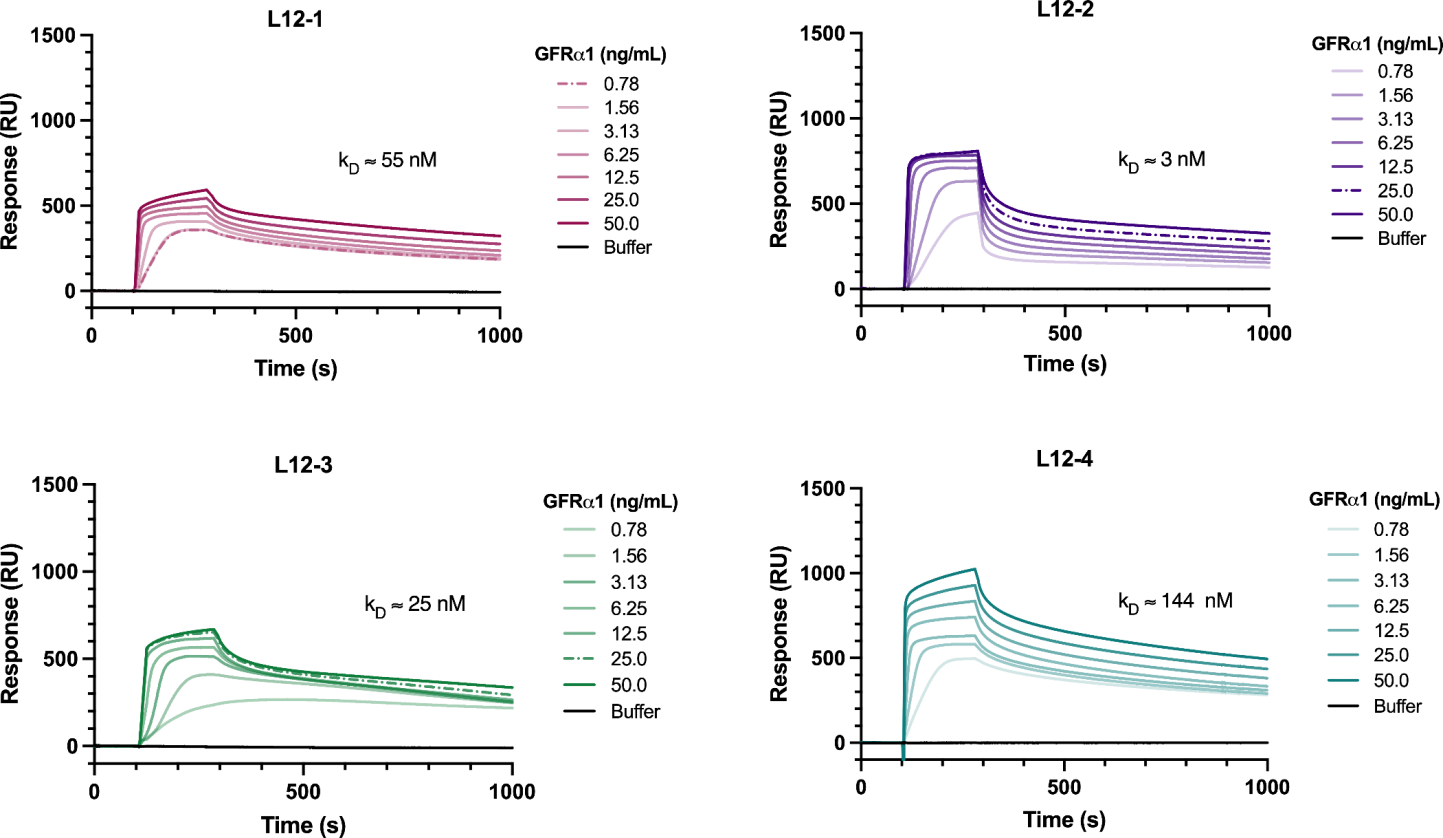
Peptides bind to GFRα1 with nanomolar affinity, determined with surface plasmon resonance (SPR). Each peptide was immobilized onto a C1 chip using amine coupling, and the GFRα1 receptor was passed over the surface at a range of concentrations (0.78–50 ng/mL). Representative sensorgrams show dose-dependent binding of the target receptor against the immobilized peptides. The equilibrium dissociation constant (*K*_D_) was calculated using steady-state affinity models, based on the binding response at equilibrium for at least five concentrations of the analyte (GFRα1), in duplicate.

Following receptor binding, GDNF is known to elicit proliferation of the SH-SY5Y neuronal-like cell line.^52^ Treatment of SH-SY5Y cells with the peptides led to an increase in phosphorylated AKT, detected with a phosphoAKT ELISA. TRO-1938, a small molecule which activates the PI3K pathway directly (EC_50_ of ∼ 60 μM for PI3Kα, based on lipid kinase activity *in vitro*), was used as a positive control.^53^ The results confirmed that at the concentrations used in this assay, the peptides activate the PI3K/AKT regenerative signaling pathway at a 75 min time point (Figure 5A).

**Figure 5:**
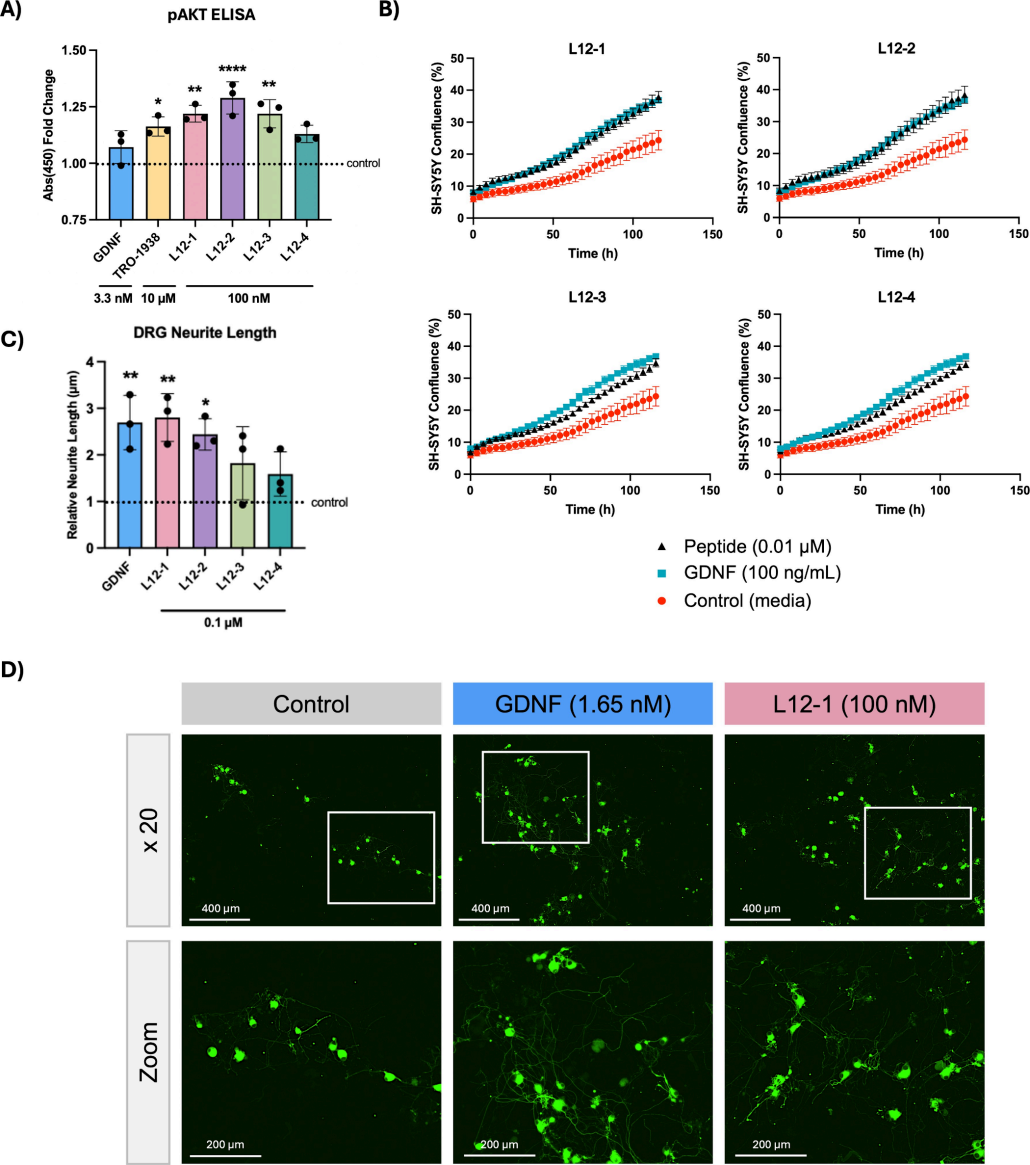
Peptides activated the PI3K signaling pathway, increased SH-SY5Y cell confluency and enhanced DRG neurite outgrowth. (A) PI3K pathway activation was measured by lysing SH-SY5Y cells treated with 3.3 nM GDNF (100 ng/mL), 10 μM TRO-1938, or 100 nM peptide for 75 min. Phosphorylated AKT was measured with a pAKT ELISA (*n* = 3 wells, mean ± SD) per condition. Statistical significance was calculated for each group compared to the untreated control condition (*A*_450 nm_ mean value = 0.840 ± 0.075), using a one-way ANOVA with a Dunnett’s multiple comparisons test, where *P ≤ 0.05, **P ≤ 0.01 and **** *P* ≤ 0.0001. (B) Representative percentage confluency graphs over time for SH-SY5Y cells treated with 3.3 nM (100 ng/mL) GDNF or 10 nM peptide. Confluency was measured using the Incucyte Live Cell Imaging System (*n* = 3 wells, mean ± SEM). (C, D) DRGs harvested from adult Sprague–Dawley rats, dissociated and cultured for 24 h and treated with 1.65 nM (50 ng/mL) GDNF or 100 nM peptide. After 48 h, cells were fixed and immunostained to detect β-III-tubulin. Cells were imaged with a ×10 objective (Zeiss) and neurite lengths were measured using ImageJ. Data is shown as average neurite length per DRG (mean ± SD) relative to the control condition (64.44 ± 44.73 μm), from 3 independent experiments, each with 3 technical replicates per condition. Statistical significance was calculated for each group compared to the untreated control condition, using a one-way ANOVA with a Dunnett’s multiple comparisons test, where *P ≤ 0.05 and **P ≤ 0.01.

Activation of the PI3K/AKT pathway is known to promote cell survival and proliferation,^54^ therefore SH-SY5Y cell confluency was measured over time with various doses of each peptide (Figure S11). Cell confluency refers to the percentage of the available surface that is covered by cells and while it does not provide specific details about cell number, proliferation or morphology it is a useful and nondestructive way to assess the behavior of a cell population over time. Treatment of SH-SY5Y cells with the peptides (10 nM) led to an increase in cell confluency to the same extent as GDNF at 3.3 nM (100 ng/mL) over 5 days, measured using the Incucyte live cell imaging system (Figure 5B). To measure their tissue regenerative potential, peptides were tested in an *in vitro* primary neuron assay to measure neurite outgrowth, using adult rat DRG neurons. The optimal concentration of GDNF for maximum neurite outgrowth was determined to be 1.65 nM (50 ng/mL), which was used as a positive control (Figure S13).^20,55^ Peptides L12–1 and L12–2 (100 nM) increased number of DRGs containing neurites 3–4-fold and DRG neurite length by 2–3-fold, compared to the untreated control condition, an increase which was statistically indistinguishable from the effect of GDNF (Figure 5C,D and Figure S14).

With the limitations associated with delivering recombinant GDNF as a therapeutic, it is a clinical imperative to develop smaller peptides that can mimic its beneficial, therapeutic effects.^11,36^ Here, four hit peptides were selected by screening the Ph.D-12 library against GFRα1, using phage display technology. All four peptides mimicked GDNF by binding to the GFRα1 receptor and demonstrated comparable *in vitro* responses in both a cell line and primary neuronal cells. As peptide mimetics of GDNF have great therapeutic potential, three other peptide mimetics have been previously identified based on the native sequence of GDNF family ligands.^37–39^ Of these, only one, a peptide dendrimer named artefin, has been confirmed to act via GFRα1 binding,^37^ and has more limited therapeutic potential than monomeric peptides due to its size and synthetic complexity. To date, this work is the first report of peptide monomers that mimic the activity of GDNF by binding to GFRα1. All four peptides identified in this study through phage display screening demonstrated therapeutic potential, with L12–1 and L12–2 as leading candidates.

The current study demonstrated that monomeric peptides selected against GFRα1 can mimic the biological activity of GDNF in SH–SY5Y cells and adult rat DRG neurons *in vitro*. However, several questions remain regarding the precise mechanism by which the peptides elicit their biological effects. Future work will therefore need to experimentally determine the binding interactions between the peptides and GFRα1, assess whether peptide engagement is sufficient to induce GFRα1 dimerization, and determine the stoichiometry of the resulting peptide-GFRα1 complexes. In addition, it will be important to clarify whether the peptides act competitively or cooperatively with endogenous GDNF, and to establish whether their signaling mechanism is dependent on RET, NCAM, or a combination of both pathways. Future work should also include kinase profiling studies to elucidate the downstream signaling pathways activated by the peptides. Addressing these mechanistic questions will be essential for understanding how the peptides activate GDNF signaling and guiding their future development.

Although peptide therapeutics can be limited by pharmacokinetic challenges, such as proteolytic instability, rapid clearance, and poor membrane permeability, these challenges present clear opportunities for future optimization. Advances in peptide synthesis and modification, and the development of novel delivery systems,^56^ offer promising strategies to enhance stability, bioavailability, and target engagement, supporting the translational potential of these GFRα1–targeting peptides.

## Conclusions

Therapeutically relevant GFRα1-targeting peptides were identified using phage display screening, with nanomolar binding affinity and comparable biological efficacy to GDNF *in vitro*. The top two peptides identified here (L12–1 and L12–2) have demonstrated the potential to aid tissue regeneration and have therapeutic potential in a wide range of clinical applications.

## Experimental Section

### General Experimental

Ph.D.-12 phage display peptide library was purchased from New England Biolabs. His-tagged human GFRα1 was purchased from Sino Biological. HisPur nickel-nitrilotriacetic acid magnetic capture beads were purchased from ThermoFisher. Peptide synthesis grade DMF was used for peptide synthesis. Peptide synthesis reagents were purchased from Novabiochem, Merck. All solvents used for HPLC were HPLC-grade and were used directly from the bottle. High resolution mass spectrometry was conducted using Q-TOF Liquid Chromatography–mass spectrometry on an Agilent AdvanceBio 6545 XT LC/Q-TOF system using a linear gradient of 3–80% B over 12 min (A = water with 0.1% formic acid and B = acetonitrile with 0.1% formic acid) with an Agilent PowerShell 120 column (3.0 × 30 mm) column and a 0.4 mL/min flow rate. The setup included a G6545XT NS QTOF and an AJS-ESI ion source and detected a mass range of 100–3000 *m*/*z*. All peptides were purified using reverse-phase HPLC on an Agilent 1260 Infinity II HPLC system with a quaternary pump and VWD detector, using a Zorbax 300SB-C18 column (5 μm, 9.4 × 250 mm, Agilent). The sample was manually injected (0.1–1.0 mL) and the instrument was run with a flow rate of 3 mL/min, column temperature of 20 °C, and detection wavelength of 214 nm. Analytical reverse-phase HPLC was conducted on an Agilent 1260 Infinity II HPLC system with a quaternary pump and VWD detector, using a Zorbax 300SB-C18 column (5 μm, 4.6 × 250 mm, Agilent). A flow rate of 1.0 mL/min, column temperature of 20 °C, injection volume of 10 μL and detection wavelength of 214 nm was set. HPLC gradients are provided in the Supporting Information. All peptides are >95% pure by HPLC. All SPR experiments were performed using a Biacore X100 at 25 °C using C1-sensor chips (Cytivia). SH-SY5Y cells and cell culture reagents were purchased from Sigma-Aldrich, unless stated otherwise.

### Phage Display

The Ph.D.-12 phage display peptide library was screened for affinity against His-tagged human GFRα1. Three rounds of solution-phase panning were carried out with HisPur nickel-nitrilotriacetic acid (Ni-NTA) magnetic capture beads.

Ni-NTA magnetic beads (5 μL, 12.5 mg/mL in 20% ethanol) were washed with 0.1% TBST buffer (50 mM Tris-HCl, 150 mM NaCl, and 0.1% (*v/v*) Tween-20, pH 7.5) and blocked for 1 h at 4 °C with a blocking buffer (1 mL, 5 mg/mL of BSA and 0.1 M NaCl, pH 8.6). Beads were collected by magnetic capture using a rare earth magnet and washed (6 × 1 mL) with 0.1% TBST, pelleting the beads with each wash. In parallel, the Ph.D.-12 phage display peptide library (5 × 10^10^ plaque forming unit (pfu)) was incubated with His-tagged human GFRα1 (300 ng) and made up to a total volume of 200 μL with 0.1% TBST. After 20 min at room temperature, the phage-receptor mixture was added to the prewashed beads and incubated at room temperature (RT) for 15 min. Using magnetic capture, the supernatant was discarded and the beads with bound phages were washed (10 × 1 mL) with 0.1% TBST. Unbound or weakly bound phages were discarded in the supernatant. Bound phages were then eluted with glycine elution buffer (1 mL, 0.2 M glycine-HCl, 1 mg/mL BSA, pH 2.2) over 10 min at RT. Ni-NTA beads were pelleted and discarded using magnetic capture and supernatant, now containing the previously bound phage, was collected and neutralized with Tris-HCl buffer (150 μL, 1 M, pH 9.1). Subtractive panning was introduced in round 2. This negative selection process aimed to remove nonspecific binders. The amplified phages were incubated with unblocked Ni-NTA beads (5 μL) for 1 h at RT. The beads were pelleted with magnetic separation and discarded. The supernatant was taken into the second round of panning using preblocked beads (as described in round 1). Tween-20 was increased from 0.1% in round 1 to 0.2% in round 2 and 0.5% in round three. The quantity of the GFRα1 receptor was decreased 10-fold to increase competition between bound phage clones. Biopanning conditions and phage titer outputs are provided in the Supporting Information.

#### Phage Amplification

Following each round of selection, phage from the eluted pool were amplified. Eluted phages were added to a diluted overnight culture of *Escherichia coli* ( *E. coli* ) ER2738 (20 mL, 1:100 in 20 mL of LB medium, OD600 0.01–0.05) in a 250 mL conical flask and incubated for 4.5 h at 37 °C with vigorous shaking. Amplified phages were separated from *E. coli* by centrifugation (12,000 × *g*, 10 min, 4 °C). The upper 80% of the supernatant (16 mL) was transferred into a new Falcon tube and 1/6 volume of 20% PEG/2.5 M NaCl (2.67 mL) was added. PEG coats the phages, increasing the density and causing the phage to precipitate out of solution overnight at 4 °C. PEG-coated phages were obtained by centrifugation (12,000 × *g*, 15 min, 4 °C). The PEG coating was removed by suspending the pellet in TBS (1 mL, 50 mM Tris-HCl, 150 mM NaCl, pH 7.5) and residual *E. coli* cells were pelleted by centrifugation (12,000 × *g*, 5 min, 4 °C). Phage-containing supernatant was placed in a new Eppendorf tube, then reprecipitated with 1/6 volume of 20% PEG/2.5 M NaCl (0.17 mL) on ice over 1 h. The supernatant was discarded and phages were released from the PEG-coating with TBS (200 μL, 50 mM Tris-HCl, 150 mM NaCl, pH 7.5). To obtain the amplified eluate, samples were centrifuged to pellet and discard any remaining insoluble matter.

#### Phage titration

Following each selection and amplification, the phage output was quantified by titration. The library M13KE cloning vector carries the lacZα gene, meaning each phage formed a blue plaque when plated on X-gal and IPTG-coated plates. Pfu can be determined by counting the number of blue plaques in a specified volume of eluate. Serial dilutions of the phage eluate were prepared to 10^2^–10^4^ and 10^9^–10^11^ for unamplified and amplified phage stock, respectively. Dilutions were prepared in a diluted overnight culture of *E. coli* (1:100 in LB medium, 200 μL) and a sample (10 μL) was mixed with top agar (3 mL, 45 °C, 10 g/L bacto-tryptone, 5 g/L yeast extract, 5 g/L NaCl, and 7 g/L Bacto-Agar) and spread over a preheated LB/IPTG/X-gal plate (37 °C). Plates were left to set, inverted and incubated overnight at 37 °C. The plate containing well-separated, countable plaques was selected for each condition and the pfu was calculated based on the number of blue plaques and dilution factor.

#### Extraction of Single-Stranded Phage DNA and Sequencing

Phage were amplified (5 h, 37 °C) and separated from *E. coli* cells by centrifugation (14,000 × *g*, 30 s). Supernatant (500 μL) from each clone was transferred into a new Eppendorf tube with 20% PEG/2.5 M NaCl solution (200 μL) to precipitate the phage over 20 min at RT. Phage were precipitated with centrifugation (14,000 × *g*, 10 min, 37 °C) and the pellet was suspended in iodide buffer (100 μL, 4 M sodium iodide, 10 mM Tris-HCl and 1 mM EDTA, pH 8.0) to denature the phage DNA into single strands. Single-stranded DNA was precipitated, and the phage protein was broken down by mixing with ethanol (250 μL) over 20 min at RT. Samples were centrifuged (14,000 × *g*, 10 min, 4 °C), washed with cold 70% ethanol and air-dried. DNA was dissolved in water (20 μL), and the concentration was measured with a NanoDrop 200 spectrophotometer (ThermoFisher Scientific). DNA was purified through a phenol/chloroform extraction.

Phage DNA from the final round of biopanning was amplified by PCR. Illumina adaptors and sample barcode were added to phage DNA by PCR. Primers, PCR reaction conditions and cycling times are provided in the Supporting Information. Illumina sequencing was carried out on a NexSeq (Dr David Baker, Quadram Institute Biosciences, Norwich UK) as previously described,^45^ and the DNA sequences were extracted with a NGS pipeline (https://github.com/taoyangwu/P3ANUT).^45^ MimoScan was used to exclude commonly identified sequences, nonspecific binders or parasitic sequences (SAROTUP, https://i.uestc.edu.cn/sarotup/cgi-bin/MimoScan.pl).^57^ BLASTp was used to inspect the sequence homology compared to relevant proteins (UniProt, https://www.uniprot.org/blast). The remaining sequences were ranked based on the number of hits and analyzed using a multiple sequence alignment tool, Clustal Omega (EBI, https://www.ebi.ac.uk/jdispatcher/msa/clustalo).^58^

### *In Silico* Alanine Scanning Mutagenesis Using BUDE Alanine Scanning (BAlaS)

BAlaS web server was used to identify “hot spot” residues that contribute to GDNF-GFRα1 binding (https://pragmaticproteindesign.bio.ed.ac.uk/balas/),^49,59^ using published crystal structures (PDB: 6Q2N).^48^ This method used an empirical free-energy force field to calculate change in free energy (ΔΔ*G*) associated with mutating each interface residue to alanine. Hot constellations consisting of 2–4 residues were screened, showing which residues cooperate in the binding pocket. Constellation ΔΔG_binding_, summed individual ΔΔG_binding_ and cooperativity were reported. The cooperativity was calculated by subtracting the summed individual ΔΔG_binding_ from the constellation ΔΔG_binding_.

### AlphaFold3 Prediction of Peptide Secondary Structure and Receptor Binding

The AlphaFold3 server (https://alphafoldserver.com)^50^ was used for peptide secondary structure prediction and peptide–protein modeling, using standard parameters. Peptide sequences were input alongside the human GFRα1 sequence (Uniprot: P56159), removing the unstructured terminal, pre- and pro-peptide regions.

### Peptide Synthesis and Characterization

Peptides were synthesized by Fmoc SPPS (Liberty Lite Automated Microwave Peptide Synthesizer, CEM) on a 0.1 mmol scale. All reagents for coupling and deprotection reactions were dissolved in DMF. Peptides were purified by preparative HPLC (>95% purity) and characterized using high-resolution mass spectrometry and high pressure liquid chromatography. Larger quantities of peptide were purchased (Peptide Protein Research LTD) for SPR analysis. Purchased peptides were >95% pure and MS data was in agreement with that obtained for the synthesized samples.

#### Coupling

The following protected *L*-amino acids were used: Fmoc-Ala-OH, Fmoc-Arg­(Pbf)–OH, Fmoc-Asn­(Trt)–OH, Fmoc-Asp­(OtBu)–OH, Fmoc-Gln­(Trt)–OH, Fmoc-Glu­(OtBu)–OH, Fmoc-Gly-OH, Fmoc-His­(Trt)–OH, Fmoc-Ile-OH, Fmoc-Leu-OH, Fmoc-Lys­(Boc)–OH, Fmoc-Met-OH, Fmoc-Phe-OH, Fmoc-Pro-OH, Fmoc-Ser­(tBu)–OH, Fmoc-Thr­(tBu)–OH, Fmoc-Trp­(Boc)–OH, Fmoc-Tyr­(tBu)–OH, Fmoc-Val-OH.

Amino acids (0.2 M) were coupled with the activator DIC (0.5 M) and activator base Oxyma (1.0 M). Coupling reactions were carried out using the default instrument microwave conditions (90 °C for 3 min), apart from histidine residues which were coupled at 50 °C for 10 min and arginine residues that were double coupled. For a 0.1 mmol scale reaction, total reaction volume was 4 mL, using 2.5 mL amino acid, 1 mL DIC and 0.5 mL Oxyma.

#### Deprotection and Cleavage

Automated Fmoc deprotection reactions were conducted with a solution of 20% *v/v* piperidine in DMF (total reaction volume 3 mL) using the default instrument microwave conditions (90 °C for 2 min). Final Fmoc deprotection and cleavage steps were completed manually in 10 mL syringes with PTFE frits with a total reaction volume of 5 mL. For deprotection, 20% *v/v* piperidine in DMF was added to a syringe containing the resin-bound peptide and agitated on a platform shaker (Heidolph Unimax 1010) at 300 rpm for 3 min then evacuated by hand. This process was repeated 5 times or until no UV signal was observed. The resin was washed with DMF (6 × 1.5 mL) to eliminate any remaining piperidine solution.

Resins were washed with DMF (3 × 1.5 mL), CH_2_Cl_2_ (3 × 1.5 mL) and diethyl ether (3 × 1.5 mL) before drying under vacuum overnight. The cleavage solution consisted of 95% TFA, 2.5% TIPS and 2.5% water (*v/v/v*). The cleavage solution (3 mL) was added to the resin and left on the shaker for 2 h. The solution was then added to cold diethyl ether (10 mL) to precipitate the peptide. Another portion of the cleavage solution (1 mL) was added to the resin and left on the shaker for 40 min, then this solution was combined with the same ether solution. The peptide suspension was centrifuged (Fisherbrand GT4) for 15 min at 4 °C and 4000 × *g*. The supernatant was removed and discarded, then the peptide was resuspended in cold diethyl ether (10 mL). This process was repeated three times in total before dissolving the peptide pellet in water for lyophilization (BUCHI L-300). The lyophilized peptide sample was dissolved in water with minimum acetonitrile (∼5 mg/mL), and filtered through a 0.22 μm PTFE syringe filter for purification by HPLC. Following purification by HPLC, peptides were salt exchanged via repeated lyophilization from HCl (10 mM). Characterization data for each peptide can be found in the Supporting Information.

#### L12–1

Peptide L12–1 (TNSHHLHHSAQY) identified from screening the Ph.D.-12 phage display peptide library against the GFRα1 receptor was synthesized using a low-loading Fmoc-Tyr­(tBu)-Wang resin (0.2–0.4 mmol/g loading), lyophilized and purified using previously described procedures. The peptide was obtained as a fluffy white powder with a yield of 11% (8.1 mg) and 100% purity by analytical HPLC (retention time = 15.09 min). *m*/*z* (HRMS, ES+) required for [C_61_H_86_N_22_O_19_] 1431.6518, found 1431.6542 (error 1.7 ppm).

#### L12–2

Peptide L12–2 (RKQHAIPLIWPA) identified from screening the Ph.D.-12 phage display peptide library against the GFRα1 receptor was synthesized using a H-Ala-2-Cl­(Trt) resin (0.72 mmol/g loading), lyophilized and purified using previously described procedures. The peptide was obtained as a fluffy white powder with a yield of 5% (3.7 mg) and 98.6% purity by analytical HPLC (retention time = 20.61 min). *m*/*z* (HRMS, ES+) required for [C_68_H_108_N_20_O_14_] 1429.8432, found 1429.8445 (error 1.3 ppm).

#### L12–3

Peptide L12–3 (VVSPDMNLLLTN) identified from screening the Ph.D.-12 phage display peptide library against the GFRα1 receptor was synthesized using Fmoc-Asn­(Trt)-Wang resin (0.4–1.0 mmol/g loading), lyophilized and purified using previously described procedures. The peptide was obtained as a fluffy white powder with a yield of 8% (5 mg) and 100% purity by analytical HPLC (retention time = 20.40 min). *m*/*z* (HRMS, ES+) required for [C_68_H_108_N_20_O_14_] 1315.6931, found 1315.6929 (error <1 ppm).

#### L12–4

Peptide L12–4 (QQRPYVQDLRLI) identified from screening the Ph.D.-12 phage display peptide library against the GFRα1 receptor was synthesized using a Fmoc-Ile-Wang resin (0.5–1.0 mmol/g loading), lyophilized and purified using previously described procedures. The peptide was obtained as a fluffy white powder with a yield of 3% (2 mg) and 98% purity by analytical HPLC (retention time = 19.07 min). *m*/*z* (HRMS, ES+) required for [C_68_H_113_N_21_O_19_] 1528.8600, found 1528.8595 (error <1 ppm).

#### L12–5

Peptide L12–5 (SGVYKVAYDWQH-NH_2_), a lower-ranked peptide from screening the Ph.D.-12 phage display peptide library against the GFRα1 receptor, was synthesized using a Rink amide resin (0.41 mmol/g loading, 0.041 mmol scale), lyophilized and purified using previously described procedures. The peptide was obtained as a fluffy white powder with a yield of 15% (9 mg) and 93% purity by analytical HPLC (retention time = 16.65 min). *m*/*z* (HRMS, ES+) required for [C_68_H_94_N_18_O_18_] 1451.7066, found 1451.7096 (error 2.1 ppm).

### Measurement of Binding Constant of Peptides with SPR

#### Immobilization

Each peptide was immobilized onto flow cell two of C1 sensor chips using a standard amine coupling (0.05 M NHS, 0.2 M EDC), with 10 mM sodium acetate (pH 5.0) running buffer. Following three start up cycles, the flow cell was washed with glycine NaOH (0.1 M), the peptide was immobilized with a contact time of 1080 s and a flow rate of 5 μL/min and then the flow cell was washed with ethanolamine hydrochloride and deionized water. Immobilization levels for each peptide are provided in the Supporting Information.

#### Kinetic Binding Experiments

Binding experiments were performed using HBS-EP+ running buffer (0.01 M HEPES, pH 7.4, 0.15 M NaCl, and 0.005% *v/v* surfactant P20) for binding analysis. The GFRα1 (analyte) was prepared at 50 μg/mL in HBS-EP+ running buffer with up to seven 2-fold serial dilutions to determine the binding affinity. Following three startup cycles, the analyte was captured onto the chip at various concentrations, with a contact time of 180 s and dissociation time of 600 s. After each concentration, the chip was regenerated with NaOH (50 mM) for 180 s, with a stabilization period 600 s. Sensorgrams were double referenced to account for any bulk refractive index changes and nonspecific binding by subtracting the blank (buffer) control and the response from the control channel. Each experimental run was repeated in duplicate. Steady state affinity was calculated using the analyte concentrations that reached equilibrium during the experiment, using Biacore X100 Evaluation Software. Steady state affinity analysis for each peptide is provided in the Supporting Information.

### SH-SY5Y Cell Culture

SH-SY5Y cells (Sigma-Aldrich) were maintained in complete media containing 15% fetal bovine serum (FBS, Sigma-Aldrich) (1:1 *v/v* Hams F12: Eagle’s Minimum Essential media, 1% nonessential amino acids, 2 mM *L*-glutamine, 1% *v/v* penicillin/streptomycin (P/S)) in a humidified incubator at 37 °C with 5% CO_2_. Cells were passaged at 80% confluency by trypsinisation (trypsin-EDTA 1X, Sigma-Aldrich), centrifugation at 100 × *g* for 5 min and resuspension in complete media. Cells were seeded at the desired density in flasks for maintenance, or into 96 well plates for further experiments.

#### Detection of AKT Phosphorylation with an ELISA

SH-SY5Y cells were seeded in 96-well plates (50,000 per well) in complete media containing 5% FBS (Sigma-Aldrich). The next day, cells were treated with the peptides (0.1 μM) and incubated for 75 min at 37 °C with 5% CO_2_. Cells were then washed with PBS and lysed (lysis buffer 6, R&D systems). Phosphorylated AKT levels were determined using the phospho-AKT­(S473) pan-specific Duoset IC ELISA (R&D Systems, DYC887BE) following the manufacturer’s instructions (n = 3 replicates per condition). Absorbance was measured at 450 nm using a Spectramax M2e microplate reader (Molecular Devices).

#### SH-SY5Y Cell Confluency Analysis

SH-SY5Y cells were seeded in 96-well plates (7,500 per well) in complete media containing 5% FBS. After 24 h, cells were treated with the peptides (10 μM, 1 μM, 0.1 μM, 0.01 μM, 0.001 μM). Confluency was measured using the Incucyte S3 Live Cell Imaging System at 4 h intervals. Percentage confluency (compared to control) was compared between peptide concentrations, 24 h post treatment (average starting confluency between three replicates = 11% ± 1.5, 20% ± 0.5 and 15% ± 2, for each independent experiment).

### Dorsal Root Ganglion (DRG) Harvest and Culture

DRG neurons were obtained from adult Sprague–Dawley rats (two female and one male, weighing 300–600 g). Rats were culled humanely in in accordance with the UK Animals (Scientific Procedures) Act 1986, approved by UCL Animal Welfare and Ethics Review Board (AWERB) (using CO_2_ asphyxiation, followed by neck dislocation). DRGs were extracted and cleaned under a dissecting microscope and placed in Dulbecco’s Modified Eagle Medium (DMEM, ThermoFisher). DRGs were incubated with 0.125% collagenase type VI (ThermoFisher) for 90 min at 37 °C with 5% CO_2_, then mechanically dissociated by trituration. The collagenase solution was removed through centrifugation (200 × *g*, 5 min) and the dissociated cells were resuspended in DMEM with 10% FBS and 1% P/S, and seeded into a T75 flask which was supplemented with 0.01 M cytosine arabinoside (Ara-C, Sigma-Aldrich) for 24 h.

#### DRG Neurite Outgrowth

Cells from DRGs were detached from the flask with Trypsin-EDTA (1X, Sigma-Aldrich), centrifuged (200 × *g*, 5 min) and resuspended in DMEM containing 10% FBS and 1% P/S. For each experiment, cells from one rat were evenly seeded across 36 wells in a 96 well plate, coated with poly-*D*-lysine. After 24 h, cells were treated with GDNF or the peptides in DMEM with P/S. After 48 h, cells were fixed with 4% paraformaldehyde (ThermoFisher) for 30 min at room temperature, washed with phosphate buffered saline (PBS, Sigma-Aldrich) and stored at 4 °C.

#### Immunocytochemistry and Image Acquisition

Fixed cells were permeabilized and blocked with PBS containing 0.25% Triton X-100, 2.5% native horse serum (NHS) and 2.5% native goat serum (NGS) for 1 h at room temperature. Neurons were then immunostained with antimouse b-III tubulin AlexaFluor 488 conjugate (1:500, Abcam) and nuclei labeled with Hoechst 33258 (1:500, Sigma-Aldrich) for 3 h at room temperature. Cells were washed with PBS and stored at 4 °C protected from light. Five images were taken per well using a ×10 objective on an inverted fluorescence microscope (Zeiss Axiovert 200M), using a standardized sampling protocol. If no neurons were located within the fixed sampling position, the field of view was shifted one field at a time until neurons were observed. The number of neurons per field was counted and the length of each neurite was measured using the NeuronJ plugin on ImageJ. Any neurites extending beyond the field or not clearly projecting from a cell body within the field, were not measured. For neurites with branches, the longest branch only was measured.

## Supplementary Material



## Notes

Additional experimental details; characterization data for peptides; Tables S1–S8; Figures S1–S9 (PDF)

## Abbreviations

AKT: protein kinase B
BDNF: brain-derived neurotrophic factor
DIC: *N,N*′-diisopropylcarbodiimide
DMEM: Dulbecco’s modified Eagle medium
DRG: dorsal root ganglion
EDC: 1-ethyl-3-(3-(dimethylamino)­propyl)­carbodiimide
ERK: extracellular signal-regulated kinase
FBS: fetal bovine serum
GDNF: glial cell line-derived neurotrophic factor
GFRα1: GDNF family receptor alpha 1
IPTG: isopropyl β-d-1-thiogalactopyranoside
LB: Luria broth
MAPK: mitogen-activated protein kinase
NCAM: neuronal cell adhesion molecule
NGS: next-generation sequencing
NHS: *N*-hydroxysuccinimide
NTA: nitrilotriacetic acid
P/S: penicillin/streptomycin
RET: rearranged during transfection tyrosine kinase receptor
SPPS: solid-phase peptide synthesis
SPR: surface plasmon resonance
