# Supplementary material for: Peptides Targeting GDNF Family Receptor Alpha 1 (GFRα1) Mimic Glial Cell Line-Derived Neurotrophic Factor (GDNF) Bioactivity
Source: J Med Chem. 2026 May 8;69(10):11914–25. doi: 10.1021/acs.jmedchem.5c03413 (PMC13224089; doi:10.1021/acs.jmedchem.5c03413)
Supplement: Supplementary file 1 [file jm5c03413_si_001.pdf]

SUPPLEMENTARY INFORMATION

Peptides targeting GDNF family receptor alpha 1 (GFRα1) mimic glial cell line-derived neurotrophic factor (GDNF) bioactivity

Emily A. Atkinson,[1,2]* Tianyang Liu,[1,2] Maria Fowler,[1,2] Poppy O. Smith,[1,2] Alethea B. Tabor,[3] Christopher J. Morris,[1] James B. Phillips,[1,2]* Rachael Dickman[1]*

[1] UCL School of Pharmacy, UCL, 29-39 Brunswick Square, London, WC1N 1AX, UK.

[2] UCL Centre for Nerve Engineering, 29-39 Brunswick Square, London, WC1N 1AX, UK.

[3] Department of Chemistry, UCL, 20 Gordon Street, London, WC1H 0AJ, UK.

* emily.atkinson@ucl.ac.uk

* jb.phillips@ucl.ac.uk

* rachael.dickman.13@ucl.ac.uk

Table of Contents

1. Phage display screening S4
2. *In silico* studies S6
3. Peptide synthesis and characterisation S9
4. Measurement of binding constant of peptides to GFRα1 with SPR S15
5. SH-SY5Y cell culture S18
6. Dorsal root ganglion (DRG) harvest and culture S20

List of Tables

Table S1: Biopanning conditions and phage titre outputs. S4

Table S2: Reagent mix for the PCR reaction. S4

Table S3: PCR cycling conditions on QuantStudio3 Real-Time PCR. S4

Table S4: Hot spots on GDNF using alaning scanning mutagensis. S6

Table S5: Hot constellations within GDNF, using alanine scanning mutagensis. S7

Table S6: Semi-preparative HPLC gradient method for peptide purification. S9

Table S7: Analytical HPLC gradient method for peptide purity detection. S9

Table S8: Peptide immobilization levels for SPR. S15

List of Figures

Figure S1: Agarose (2%) gel electrophoresis of DNA obtained from the Ph.D-12™ phage library with a low molecular weight DNA ladder S5

Figure S2: Sequence analysis of the top 20 ranked peptides from phage display screening. S5

Figure S3: Alphafold3 peptide and protein structure prediction S8

Figure S4: Chemical structure of L12-1, HRMS spectrum and analytical HPLC chromatogram S10

Figure S5: Chemical structure of L12-2, HRMS spectrum and analytical HPLC chromatogram S11

Figure S6: Chemical structure of L12-3, HRMS spectrum and analytical HPLC chromatogram S12

Figure S7: Chemical structure of L12-4, HRMS spectrum and analytical HPLC chromatogram S13

Figure S8: Chemical structure of L12-5, HRMS spectrum and analytical HPLC chromatogram S14

Figure S9: SPR steady state affinity analysis (L12-1 to L12-4). S16

Figure S10: Controls for SPR experiments S17

Figure S11: Dose response for peptides on SH-SY5Y cell confluency S18

Figure S12: Representative phase contrast micrographs dose response for L12-1 and GDNF on SH-SY5Y cell confluency. S19

Figure S13: The optimal concentration of GDNF to induce neurite outgrowth was 50 ng/mL after a 48-hour treatment. S20

Figure S14: Representative micrographs of adult rat DRG neurons treated with L12-2, L12-3 and L12-4, an extension of Figure 5. .... S20

Figure S15: Number of DRGs with neurites, a secondary outcome measure for Figure 5. .... S21

Figure S16: Dose-dependent response of L12-1 on DRGSs and representative micrographs. S21

1. Phage display screening

DNA Amplification with PCR

Illumina adaptors and sample barcode were added to phage DNA by PCR with the following primers:

L1 5’-TCGTCGGCAGCGTCAGATGTGTATAAGAGACAG-NKKN[BAR]TATTCTCACTCT-3’, where BAR = NKKN GTA TAT TCT CAC TCT.

R1 5’-GTCTCGTGGGCTCGGAGATGTGTATAAGAGACAG-NKKN[BAR]CGAACCTCCACC-3’, where BAR = NKKN GTA CGA ACC TCC ACC.

PCR reaction mixtures were assembled as shown in Table S2 and thermally cycled on a QuantStudio 3 Real-Time PCR System (Applied Biosystems™) as shown in Table S3.

Table S1: Biopanning conditions and phage titre outputs.

Biopanning Round	GFRα1 (μg)	TBST (tween %)	Unamplified phage (pfu)	Amplified phage (pfu)	Negative Selection
1	0.3	0.1	2.2×10^{5}	8.8×10^{11}	N
2	0.3	0.2	2.4×10^{6}	4.2×10^{11}	Y
3	0.03	0.5	1.6×10^{9}	*N/A*	N

Table S2: Reagent mix for the PCR reaction.

Reagent	Volume per sample (μL)
5 x Platinum SuperFi II buffer	10
10 mM dNTPs	1
10 μM L1 Primer	5
10 μM L2 Primer	5
Platinum SuperFi II DNA polymerase	0.5
Phage DNA (200 ng)	14 – 22
Nuclease free H_2O	made up to 50

Table S3: PCR cycling conditions on QuantStudio3 Real-Time PCR System (Applied Biosystems).

Step	Temperature (°C)	Time (sec)	Cycle number
Initial denaturation	98	30	1
PCR: denaturing and annealing	98	10	34
	60	20	
	72	30	
Terminal extension	72	5	1

Agarose gel electrophoresis

PCR products and low molecular weight DNA ladder were each mixed with a gel loading dye 6X (1μL dye, 5 μL phage DNA in one sample and 1 μL dye, 2.5 μL DNA ladder in the other). Agarose (2%) gel electrophoresis was run with 2 μL of each sample/lane at 80 V for approximately 30 minutes.

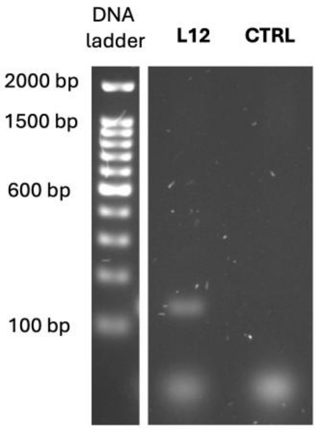

Figure S1: Agarose (2%) gel electrophoresis of DNA obtained from the Ph.D-12™ phage library with a low molecular weight DNA ladder. A band in the L12 lane (approx. 170 bp) corresponds to the amplified DNA and a fainter band in both the L12 and control lane shows the presence of a primer dimer.

Peptide Sequence Analysis

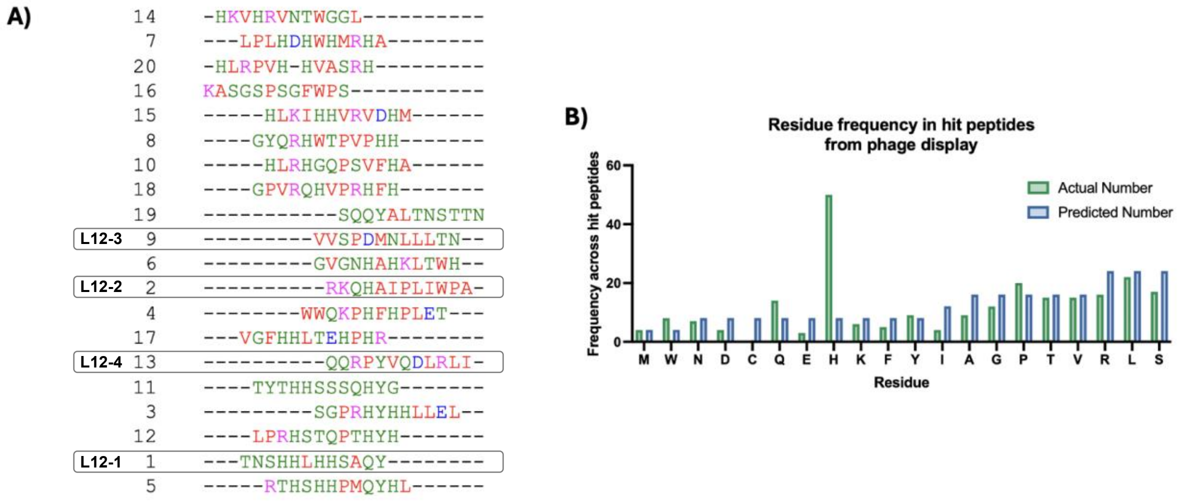

Figure S2: Sequence analysis of the top 20 ranked peptides from phage display screening. (A) Multiple sequence alignment, highlighting the alignment of His, Trp, Ser, Pro, Arg, Tyr and Leu residues across the top 20 sequences. The four selected peptides are highlighted. (B) Residue frequency in the top 20 ranked peptides compared to the predicted number of residues based on the number of possible codons per residue. Most notable is the very high frequency of His in the top 20 peptides.

2. ***In silico* studies**

Alanine scanning mutagenesis using BUDE Alanine Scanning (BAlaS)

Table S4: Hot spots on GDNF when binding to GFRα1, determined using computational alanine scanning mutagensis (PDB: 6Q2N). The table shows hot spots from the two key finger regions of GDNF (highlighted in red and yellow respectively) with a $\Delta\Delta G_{binding}$ > 0.5 kJ/mol, when mutated to alanine.

Amino Acid in GDNF	Mutated to Alanine ΔΔG (kJ/mol)
His124	1.5
Thr136	1.5
Glu138	8.6
Glu139	5.7
Leu140	0.9
Ile141	3.3
Arg143	0.9
Asp185	2.4
Asp186	2.9
Asp187	0.8
Leu188	2.2
Ser189	2.1
Phe190	1.7
Leu191	5.8
Asp193	1.9
Leu195	5.5
Val196	1.9
**Tyr197**	**20.7**
Ile199	**6.2**
His203	0.7

Table S5: Hot constellations containing residues within GDNF that cooperate when binding to GFRα1, with a cooperativity score score ≥ 0.5 kJ/mol, using alanine scanning mutagensis (PDB: 6Q2N). Residues in finger one of GDNF are labelled in red and in finger two, yellow.

No.	Constellation	Constellation ΔΔG (kJ/mol)	Summed Individual ΔΔGs (kJ/mol)	Cooperativity (kJ/mol)
		**4 Amino Acids**		
1	Glu138, Glu139, Leu191, Tyr197	43.1	40.8	2.2
2	Glu138, Leu191, Leu195, Try197	43.0	40.6	2.4
3	Glu138, Glu139, Leu191, Tyr197, Ile199	42.5	41.3	1.2
4	Glu139, Leu191, Leu195, Tyr197	41.0	37.7	3.3
5	Glu138, Glu139, Leu195, Tyr197	41.0	40.5	0.5
6	Glu139, Leu191, Tyr197, Ile199	40.4	38.4	2.1
7	Leu191, Leu195, Tyr197, Ile199	40.3	38.2	2.1
		**3 Amino Acids**		
8	Glu138, Leu191, Tyr197	37.6	35.1	2.5
9	Glu138, Glu139, Tyr197	35.6	35.0	0.6
10	Glu138, Leu195, Tyr197	35.6	34.8	0.8
11	Glu139, Leu191, Tyr197	35.6	32.2	3.4
12	Glu139, Leu195, Tyr197	33.5	31.9	1.7
13	Glu139, Tyr197, Ile199	33.0	32.6	0.4
14	Glu139, Leu191, Leu195	17.8	17.0	0.8
		**2 Amino Acids**		
15	Glu138, Tyr197	30.2	29.3	0.9
16	Leu191, Tyr197	30.1	26.5	3.5
17	Glu139, Tyr197	28.1	26.4	1.8
18	Leu195, Tyr197	28.0	26.2	1.8
19	Tyr197, Ile199	27.4	26.9	0.6
20	Glu139, Leu191	12.4	11.5	0.9
21	Leu191, Leu195	12.3	11.3	1.0
22	Glu139, Leu195	12.2	11.2	1.1

AlphaFold3 Secondary Structure and Binding Predictions

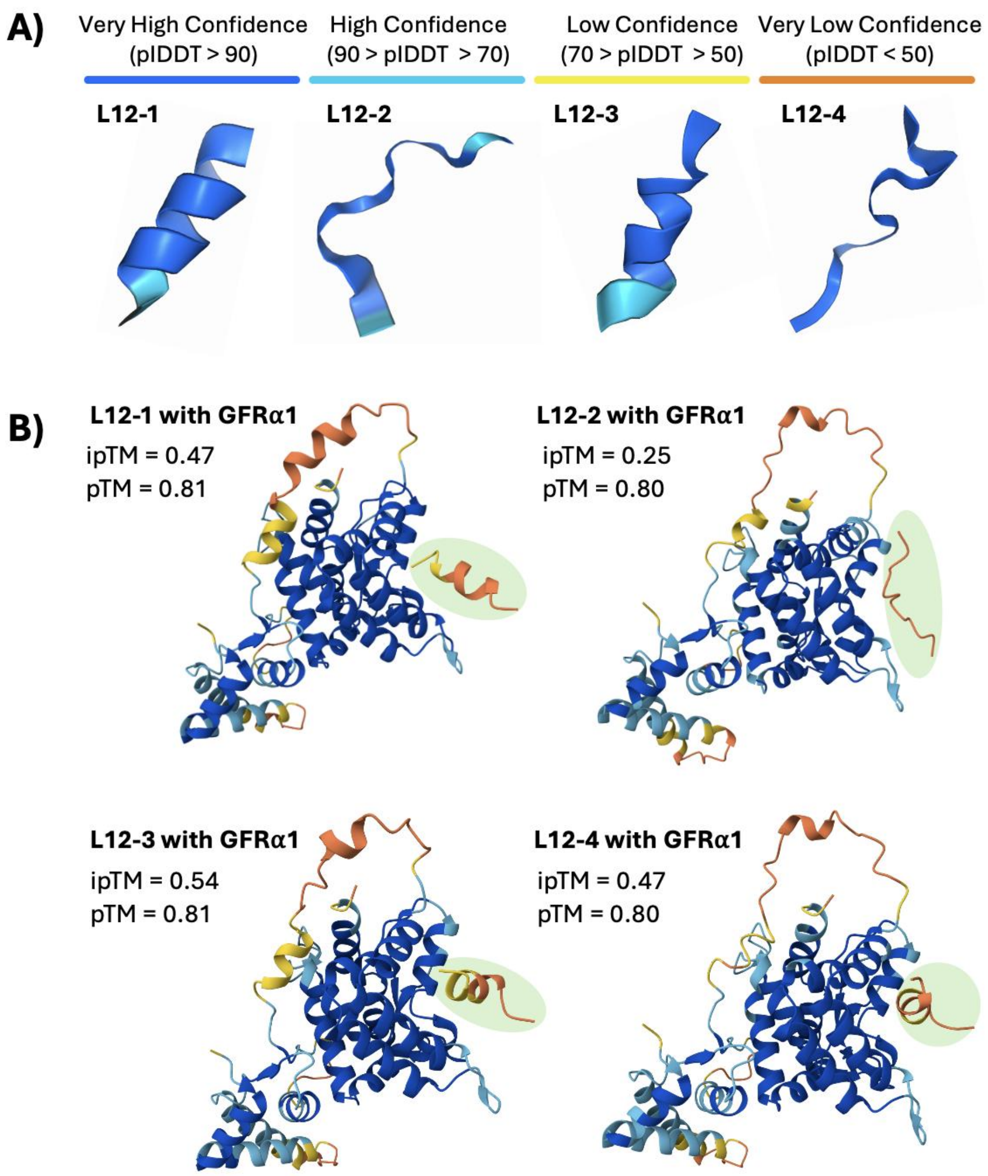

Figure S3: AlphaFold3 peptide and protein structure prediction. (A) The structure of each peptide (L12-1, L12-2, L12-3 and L12-4) was predicted with a very high or high confidence score using a per atom confidence metric (pIDDT). (B) The binding interface between of each peptide and its target receptor GFRα1 resulted in a low interface predicted template modelling score (ipTM), despite showing a high predicted template modelling (pTM) score for the prediction of each structure individually. Green circles are used to highlight the peptide in each case.

3. Peptide synthesis and characterisation

Gradients for high-performance liquid chromatography

Table S6: Semi-preparative HPLC gradient method for peptide purification.

Time (min)	Mobile Phase (%)	
	H_2O (0.1% TFA)	MeCN (0.1% TFA)
0	95	5
3	95	5
25	5	95
27	5	95
28	95	5
30	95	5

Table S7: Analytical HPLC gradient method for peptide purity detection.

Time (min)	Mobile Phase (%)	
	H_2O (0.1% TFA)	MeCN (0.1% TFA)
0	95	5
3	95	5
48	5	95
49	5	95
50	95	5
52	95	5

L12-1

A)

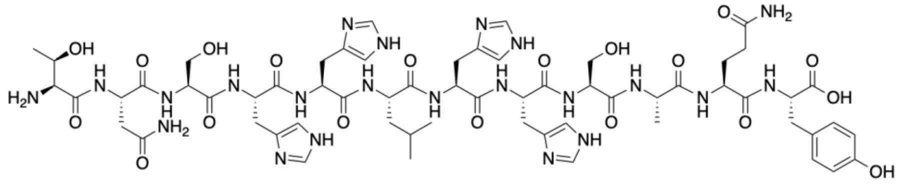

B)

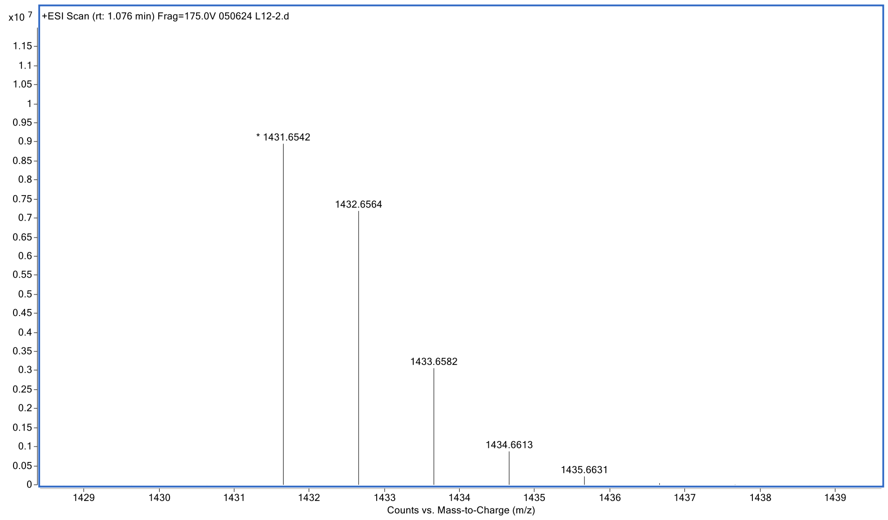

C)

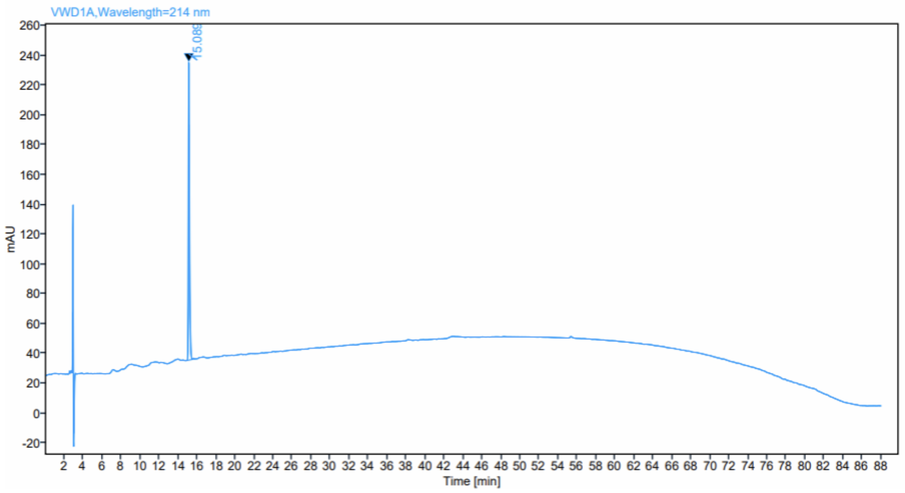

Figure S4: (A) Chemical structure of L12-1, (B) HRMS spectrum and (C) analytical HPLC chromatogram.

L12-2

A)

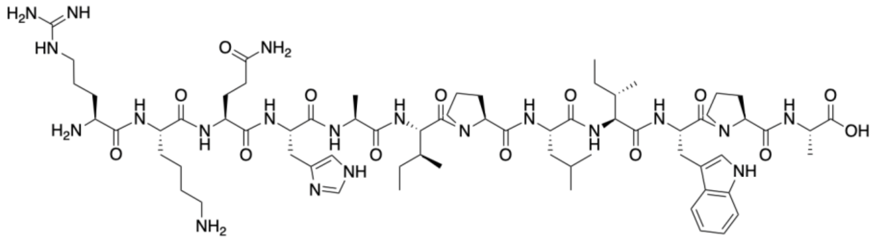

B)

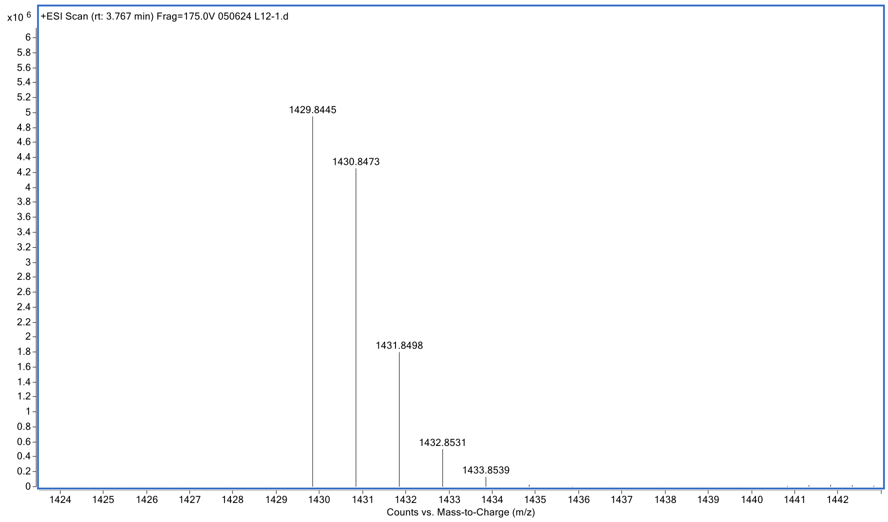

C)

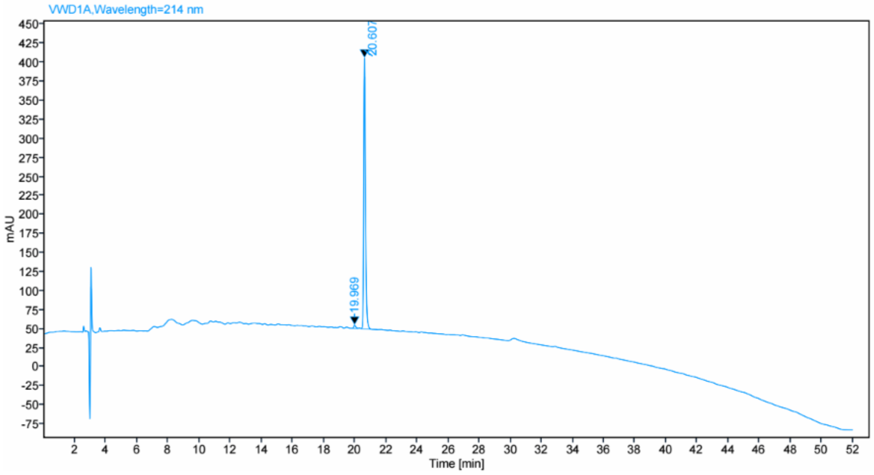

Figure S5: (A) Chemical Structure of L12-2, (B) HRMS spectrum and (C) analytical HPLC chromatogram.

L12-3

A)

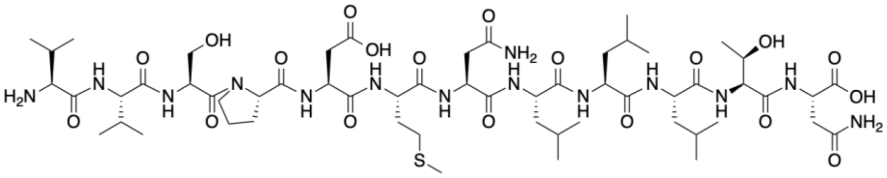

B)

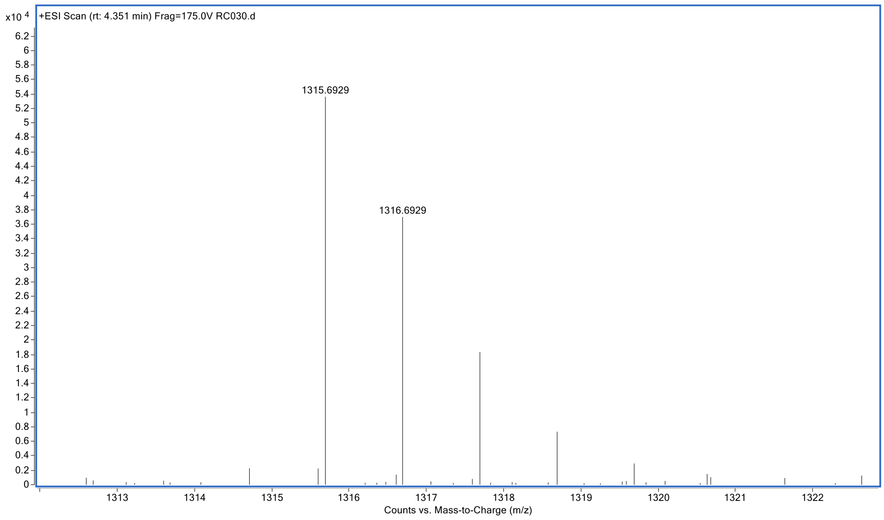

C)

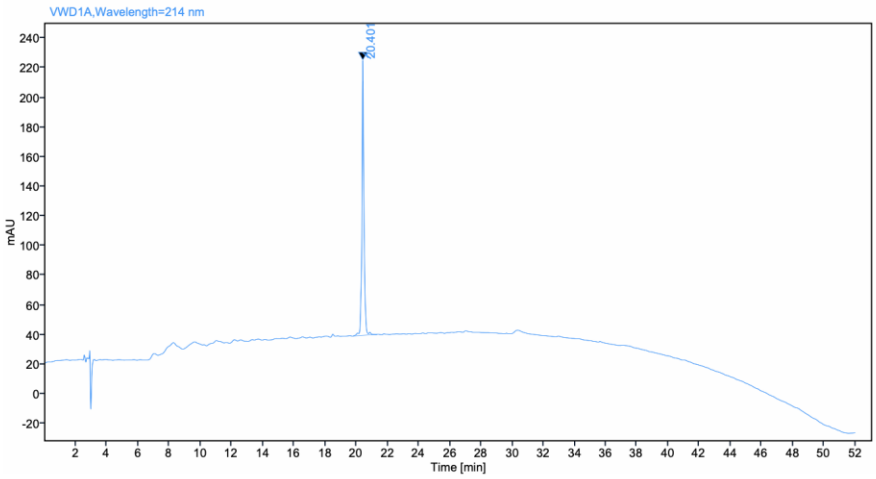

Figure S6: (A) Chemical structure of L12-3, (B) HRMS spectrum and (C) analytical HPLC chromatogram.

L12-4

A)

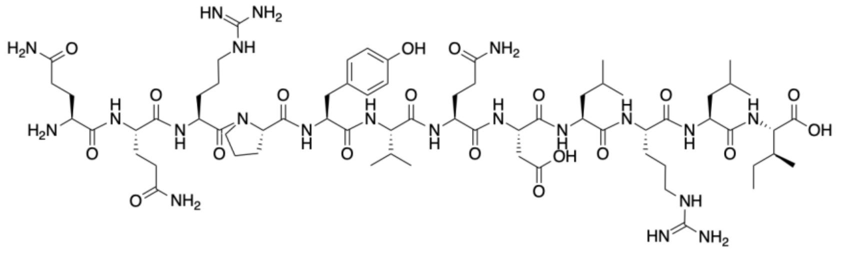

B)

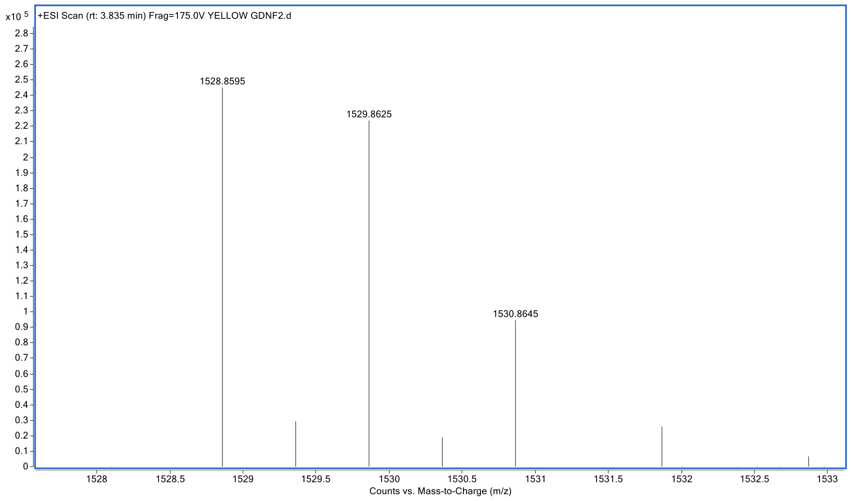

C)

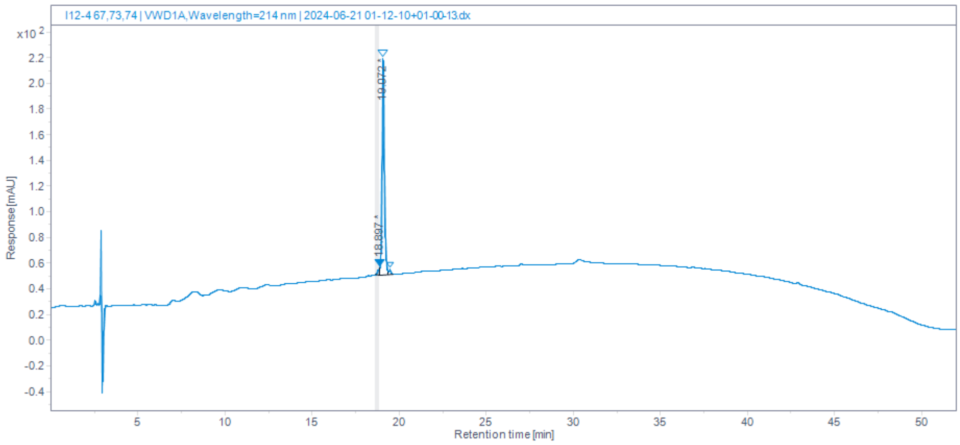

Figure S7: (A) Chemical structure of L12-4, (B) HRMS spectrum and (C) analytical HPLC chromatogram.

L12-5

A)

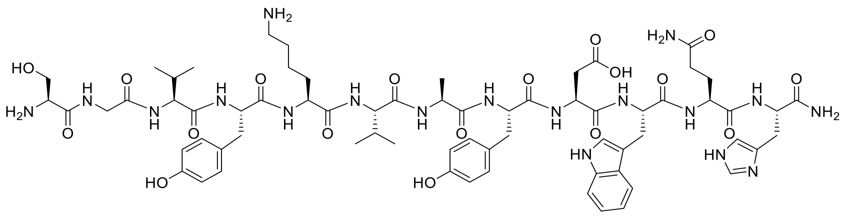

B)

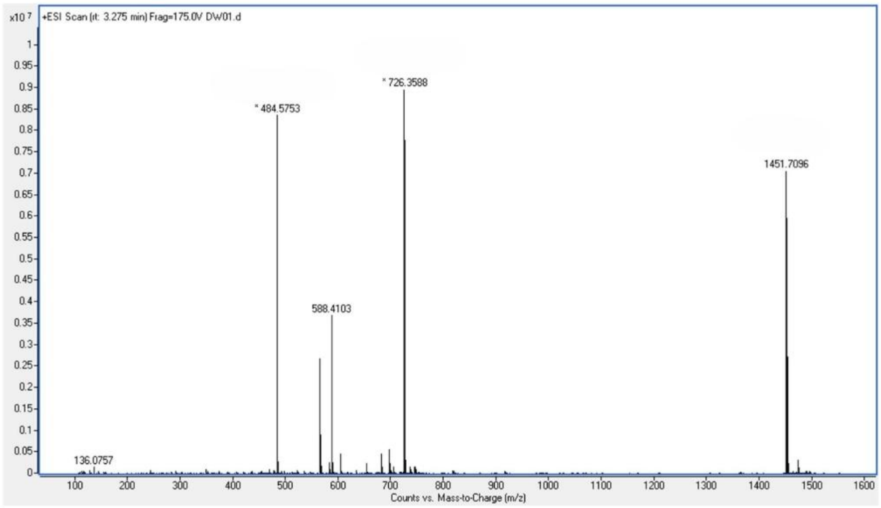

C)

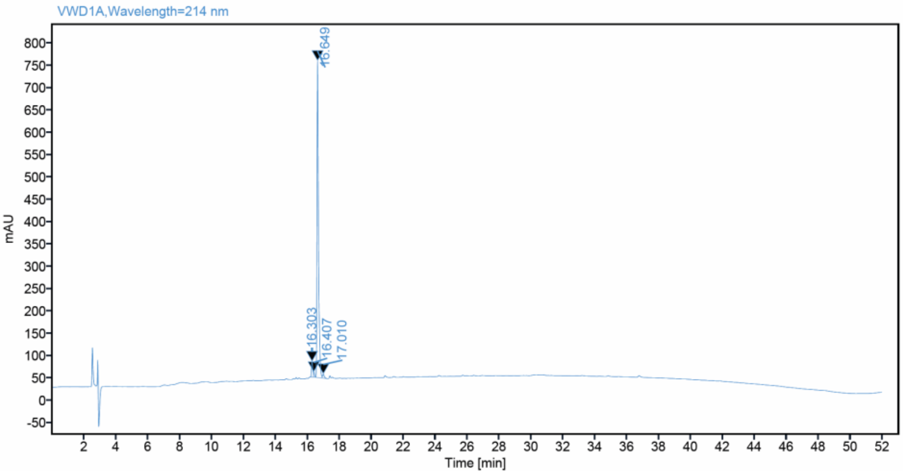

Figure S8: (A) Chemical structure of L12-5, (B) HRMS spectrum and (C) analytical HPLC chromatogram.

4. Measurement of binding constant of peptides to GFRα1 with SPR

Immobilization

Table S8: Peptide immobilization levels for SPR.

Captured Peptide (concentration)	Response Bound (RU)	Final Response (RU)
L12-1 (1000 μM)	224.7	601.1
L12-2 (1000 μM)	50.0	497.6
L12-2 (500 μM)	14.6	152.9
L12-3 (500 μM)	94.1	385.7
L12-4 (500 μM)	163.5	351.1
L12-5 (500 μM)	21.7	176.6

Kinetic binding experiments

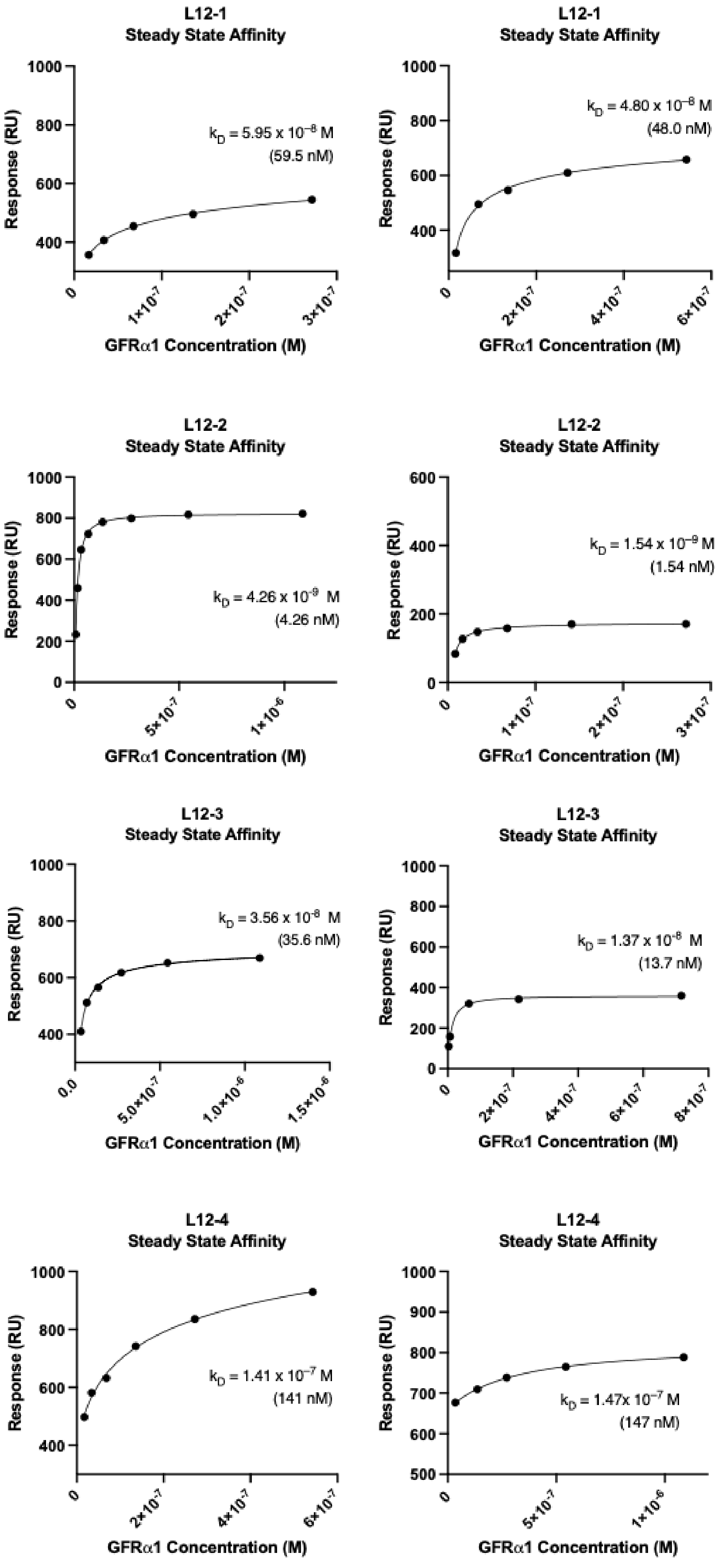

Peptide	Receptor	K_D (M)	SE (KD)	Rmax (RU)	SE (Rmax)	offset (RU)	SE (offset)	Chi² (RU^2)
L12-1	GFRα1-His	5.95E-08	1.70E-08	302.5	15	292.6	21	46.7
L12-1	GFRα1-His	4.80E-08	1.4E-08	501.8	37	501.8	188.3	214
L12-2	GFRα1-His	4.26E-09	6.90E-10	1790.5	1.90E+02	-959.7	1.90E+02	74.4
L12-2	GFRα1-His	1.54E-09	1.20E-09	585.6	3.90E+02	-411.6	3.90E+02	5.53
L12-3	GFRα1-His	3.56E-08	1.00E-08	527.7	72	155.4	77	66.6
L12-3	GFRα1-His	1.37E-08	3.00E-09	299.2	13	66.43	66.4	59.8
L12-4	GFRα1-His	1.409E-07	1.2E-08	625.6	12	430.1	9.4	33.5
L12-4	GFRα1-His	1.47E-07	3.4E-08	722.1	35	541.3	42	347

Figure S9: Results of steady state affinity analysis. SPR kinetic experiments were completed in duplicate. Concentrations of GFRα1 were not included if equilibrium was not reached with the experiment.

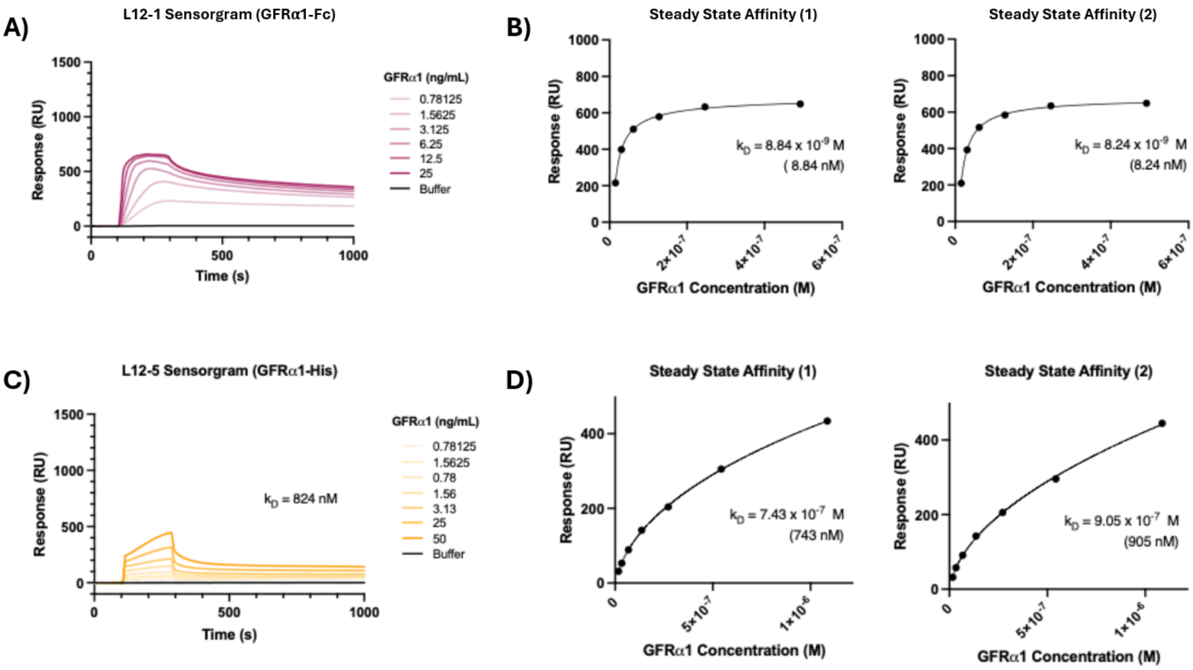

Peptide	Receptor	K_D (M)	SE (KD)	Rmax (RU)	SE (Rmax)	offset (RU)	SE (offset)	Chi² (RU^2)
L12-1	GFRα1-Fc	8.84E-09	2.0E-9	1237	1.8E+02	-568.1	1.8E+2	64.2
L12-1	GFRα1-Fc	8.235E-9	1.3E-9	1324	1.40E+2	-652.8	1.4E+2	31.5
L12-5	GFRα1-His	7.43E-07	1.20E-07	682.1	49	25	7.3	84.5
L12-5	GFRα1-His	9.05E-07	2.30E-07	746.5	93	31.2	10	179

Figure S10: Controls for SPR experiments. A) Representative sensorgram and B) steady state affinity for GFRα1-Fc screened against L12-1, to confirm the peptide was binding to the GFRα1 receptor and not the His-tag. C) Representative sensorgram and D) steady state affinity for GFRα1-His screened against L12-5, a lower-ranking peptide identified in the phage display experiment, expected to have weaker binding.

5. SH-SY5Y cell culture

SH-SY5Y cell confluency analysis

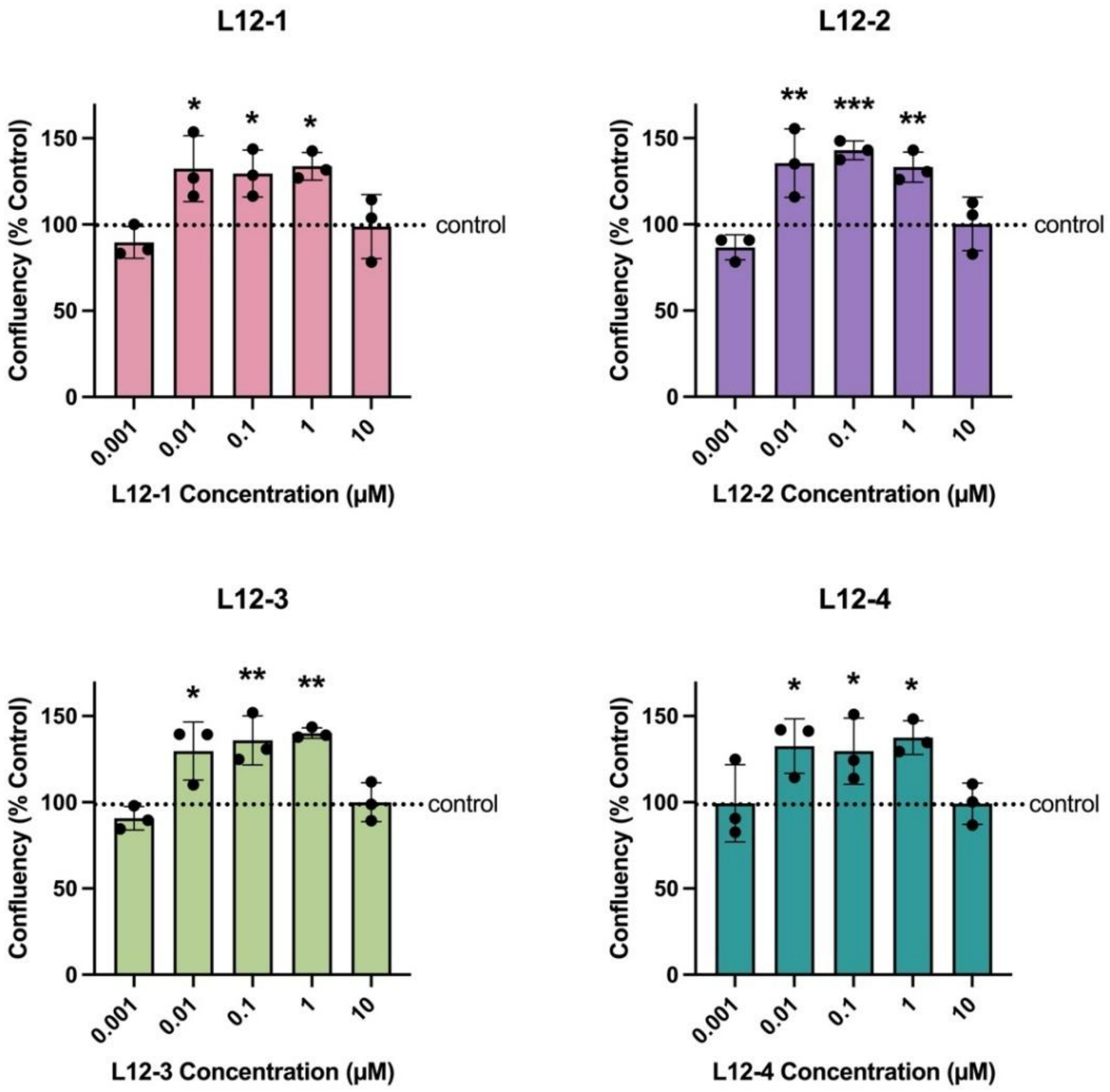

Figure S11: Effect of peptides on the confluency of SH-SY5Y cells, 24 hours after treatment. Percentage confluency of SH-SY5Y cells (passage 11-30) treated with media (control) or peptides (0.001-10 μM). Confluency was measured using images captured on the Incucyte™ S3 Live Cell Imaging System (**Figure S10**). All graphs show data as mean ± SD, from 3 independent experiments, each with 3 independent wells per condition. Statistical significance, compared to the control condition, was determined using a one-way ANOVA with a Dunnett's multiple comparisons test, where *P ≤ 0.05, **P ≤ 0.01 and ***P ≤ 0.001.

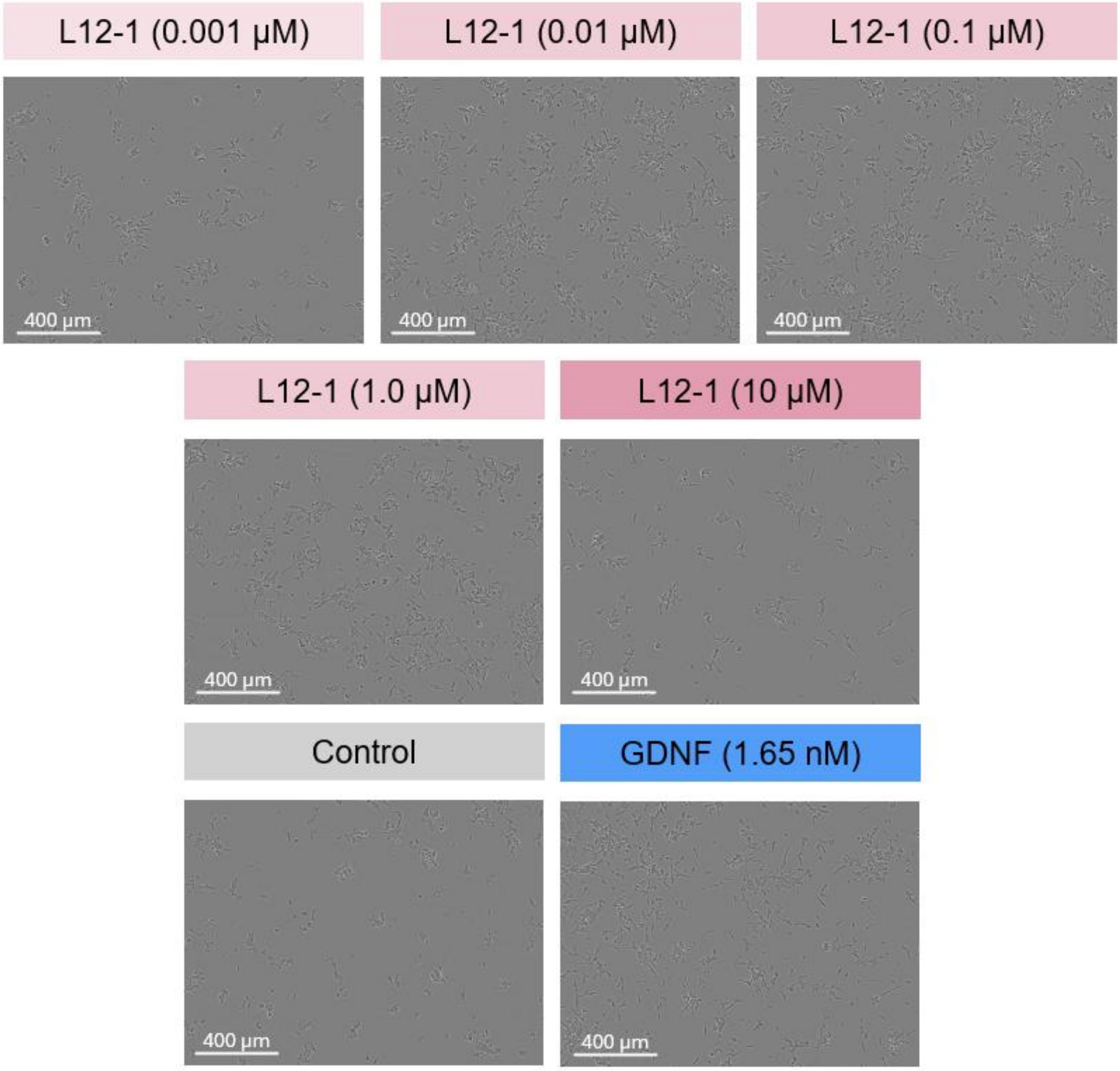

Figure S12: Representative phase contrast micrographs of SH-SY5Y cells treated with GDNF, L12-1 and cell culture media (control). Images were taken using the Incucyte™ S3 Live Cell Imaging System at x10 magnification, 24 hours after treatment.

6. Dorsal root ganglion (DRG) harvest and culture

Immunocytochemistry and image acquisition

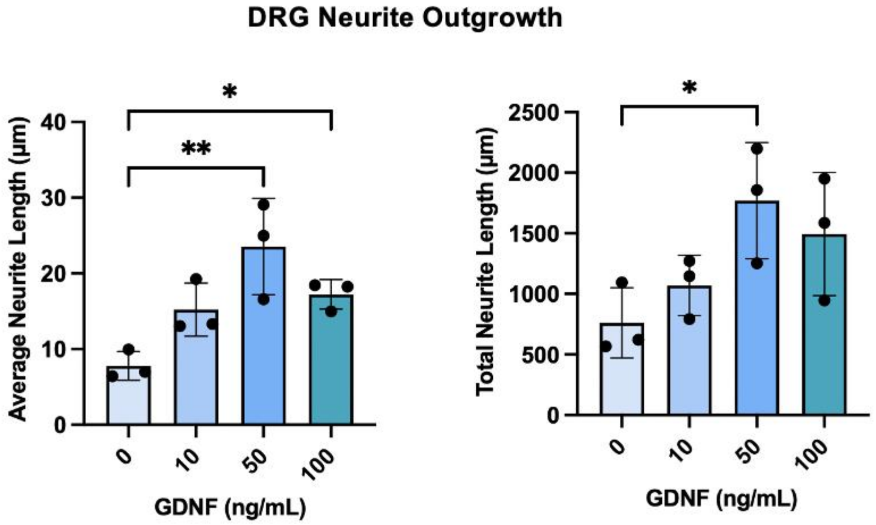

Figure S13: The optimal concentration of GDNF to induce neurite outgrowth was 50 ng/mL after a 48-hour treatment. Adult rat DRG neurons were harvested and cultured with GDNF (10, 50 or 100 ng/mL). All graphs show data as mean ± SD, from 3 independent experiments, each with 3 independent wells per condition. Statistical significance, compared to the control condition, was determined using a one-way ANOVA with a Dunnett's multiple comparisons test, where $*P \leq 0.05$ and $**P \leq 0.01$.

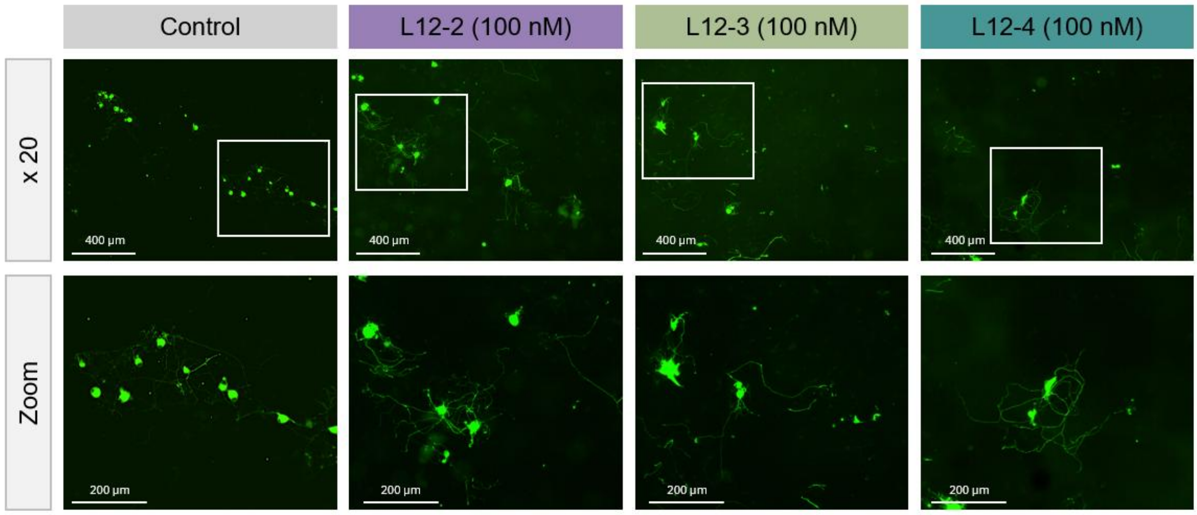

Figure S14: Representative micrographs of adult rat DRG neurons treated with L12-2, L12-3 and L12-4, an extension of Figure 5. DRG micrographs are representative images, showing DRG cell bodies and neurites stained with β-III-tubulin (green).

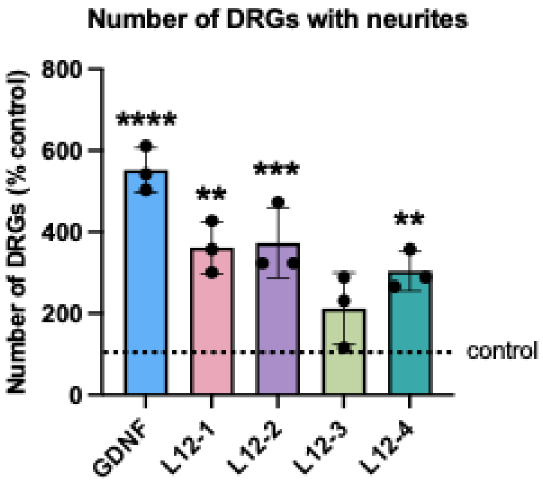

Figure S15: Number of DRGs with neurites, a secondary outcome measure for Figure 5. Statistical significance, compared to the control condition, was determined using a one-way ANOVA with a Dunnett's multiple comparisons test.

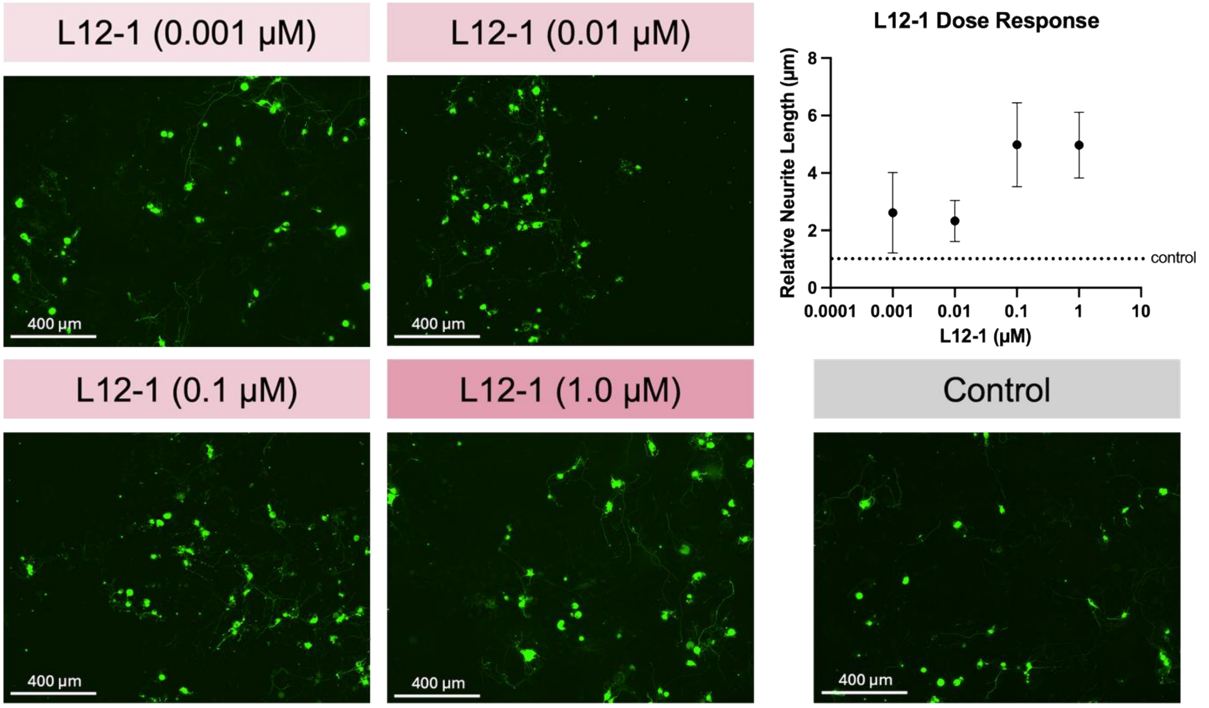

Figure S16: Dose-dependent response of L12-1 on DRGs and representative micrographs. Adult rat DRG neurons were harvested and cultured with increasing concentrations of L12-1 (0.001, 0.01, 0.1 and 1.0 μM). The dose response graph shows data as mean ± SD, from 3 independent experiments, each with 3 independent wells per condition. DRG micrographs are representative images, showing DRG cell bodies and neurites stained with β-III-tubulin (green).
